# High-resolution hybrid micro-CT imaging pipeline for mouse brain region segmentation and volumetric morphometry

**DOI:** 10.1371/journal.pone.0303288

**Published:** 2024-05-23

**Authors:** Rohan Nadkarni, Zay Yar Han, Robert J. Anderson, Alex J. Allphin, Darin P. Clark, Alexandra Badea, Cristian T. Badea

**Affiliations:** Quantitative Imaging and Analysis Lab, Department of Radiology, Duke University Medical Center, Durham, NC, United States of America; Kerman University of Medical Sciences, ISLAMIC REPUBLIC OF IRAN

## Abstract

**Background:**

Brain region segmentation and morphometry in humanized apolipoprotein E (*APOE*) mouse models with a human *NOS2* background (*HN*) contribute to Alzheimer’s disease (AD) research by demonstrating how various risk factors affect the brain. Photon-counting detector (PCD) micro-CT provides faster scan times than MRI, with superior contrast and spatial resolution to energy-integrating detector (EID) micro-CT. This paper presents a pipeline for mouse brain imaging, segmentation, and morphometry from PCD micro-CT.

**Methods:**

We used brains of 26 mice from 3 genotypes (*APOE22HN*, *APOE33HN*, *APOE44HN*). The pipeline included PCD and EID micro-CT scanning, hybrid (PCD and EID) iterative reconstruction, and brain region segmentation using the Small Animal Multivariate Brain Analysis (SAMBA) tool. We applied SAMBA to transfer brain region labels from our new PCD CT atlas to individual PCD brains via diffeomorphic registration. Region-based and voxel-based analyses were used for comparisons by genotype and sex.

**Results:**

Together, PCD and EID scanning take ~5 hours to produce images with a voxel size of 22 μm, which is faster than MRI protocols for mouse brain morphometry with voxel size above 40 μm. Hybrid iterative reconstruction generates PCD images with minimal artifacts and higher spatial resolution and contrast than EID images. Our PCD atlas is qualitatively and quantitatively similar to the prior MRI atlas and successfully transfers labels to PCD brains in SAMBA. Male and female mice had significant volume differences in 26 regions, including parts of the entorhinal cortex and cingulate cortex. *APOE22HN* brains were larger than *APOE44HN* brains in clusters from the hippocampus, a region where atrophy is associated with AD.

**Conclusions:**

This work establishes a pipeline for mouse brain analysis using PCD CT, from staining to imaging and labeling brain images. Our results validate the effectiveness of the approach, setting a foundation for research on AD mouse models while reducing scanning durations.

## Introduction

Morphometry from ex vivo brain imaging in mouse models can improve our understanding of neurodegenerative disorders such as Alzheimer’s disease (AD) and help us evaluate treatments. The typical choice of imaging modality for these studies, MRI, provides excellent contrast but is expensive and requires long scan times [1]. Contrast-enhanced X-ray micro-CT brain imaging can improve the feasibility of large-scale mouse brain morphometry studies because it provides images with 2 times the spatial resolution of MRI while reducing scan time per brain by more than an hour.

Contrast-enhanced imaging using micro-CT is an emerging technique that employs staining solutions with high atomic number (Z) elements to enhance contrast in soft tissues of biological samples [2, 3]. This technique has been used to analyze a wide variety of samples, including developing cartilage in mice [4–7] palate development [8, 9], congenital heart and kidney defects [10] and even noninvasive observation of human embryos [11].

Furthermore, contrast-enhanced micro-CT can be used to provide 3D information about inner brain structures without the need for sectioning, thereby avoiding artifacts from brain deformation or missing tissue. Micro-CT imaging has been used to visualize the vascular system of mouse brains filled with radio-opaque silicone rubber Microfil [12–17], localize cerebral ischemia [18, 19], test the efficiency of different micro-CT contrast agents [20–23], analyze specific brain structures [24], and image rat models of Alzheimer’s disease [25].

Micro-CT provides useful anatomic imaging at high spatial resolution but is limited in terms of contrast resolution by current use of energy-integrating detectors (EID) [26]. There is a clear need to improve CT and micro-CT imaging by utilizing photon-counting detectors (PCDs) [27]. To elucidate the advantages of the PCD for contrast-enhanced micro-CT, let us briefly discuss the differences between the EID and the PCD. The EID uses a scintillator to convert x-ray photons to light and detects this light to measure total energy deposited. PCDs directly convert x-ray photons into electrical signals and sort them into energy bins. Due to this difference in detection mechanism, a PCD scan provides higher spatial resolution than an EID scan at the same radiation dose [27]. Low-energy photons contribute more to image contrast for the PCD, which improves the contrast-to-noise ratio in iodine-enhanced scans. Another advantage is that high-energy thresholds of the PCD are much less affected by calcium blooming and beam hardening artifacts because these effects are caused by attenuation of low-energy photons in bone or metal [27]. Finally, the PCD is advantageous for applications that require multi-energy CT and material decomposition because it allows simultaneous acquisition of data sets at multiple energy thresholds [27].

Although the PCD has several advantages over the EID, it also has unique challenges. These include effects such as charge sharing, pulse pileup, and K-escape that distort the spectral response of the PCD [28]. PCDs with multiple semiconductor tiles can have inconsistent spectral response between tiles that leads to low frequency band artifacts in the projection domain and concentric ring artifacts in the image domain [29]. The band artifacts are typically resolved by applying correction functions in the projection domain that are calibrated using materials with known attenuation and thickness such as acrylic and aluminum plates [29] or a water phantom [30]. Past attempts to address spectral distortions include multiplicative corrections calibrated with aluminum and polyoxymethylene plates of known thickness [31], mathematical models of spectral distortion that can be incorporated into iterative reconstruction [32], and deep learning approaches using training data from PCD simulations [33]. Another strategy to reduce the adverse effects of PCD spectral distortions is to acquire PCD and EID scans of the same subject and perform hybrid iterative reconstruction using both sets of projections [34].

Our group has advanced photon-counting CT (PCCT) by building preclinical prototypes and demonstrating their value in cancer [35] and cardiac [36] studies in mice. In this study, we present a novel approach for ex vivo micro-CT imaging of mouse brains using high resolution hybrid (PCD and EID) micro-CT. We compare the performance of PCCT to energy-integrating CT in terms of image quality and accuracy in reconstructions of imaging phantoms and contrast-enhanced mouse brains. We utilize a unique combination of multiplicative band artifact removal and hybrid iterative reconstruction to address PCD artifacts. Then, we demonstrate our approach for segmentation of mouse brain regions using PCD images from hybrid iterative reconstruction. This approach transfers brain region labels to individual PCD mouse brain images via registration to our new PCD CT atlas. We use the results from this atlas-based brain region segmentation to perform morphometric analysis by genotype and sex in mouse models representing different levels of risk for late onset AD. To our knowledge, this study is the first to successfully implement a contrast-enhanced hybrid micro-CT based approach for brain region segmentation and analysis.

## Materials and methods

We employed a combination of novel animal models, meticulous brain staining protocols, advanced CT imaging and segmentation techniques, and rigorous statistical analyses to delve deep into brain morphometric analyses. The following sections describe our methodology, from the selection of animal models to the analytical assessments we performed.

### Animals

Our study used brains from the same mouse strains described in prior work [37] that express human apolipoprotein E (*APOE*) genotypes on a human *NOS2* background. A humanized, inducible *NOS2* background (*HN*) further increases the relevance of mouse models to human brain aging associated risk by making the innate immune system of the mouse more similar to that of humans. The *APOE4* allele is known to be the greatest genetic risk factor for developing AD in humans, while *APOE3* has a neutral effect and *APOE2* decreases risk of AD [38, 39]. Our mouse population consists of homozygotes for each of the major human *APOE* alleles, including 8 *APOE22HN* mice (5 male, 3 female), 9 *APOE33HN* mice (6 male, 3 female), and 9 *APOE44HN* mice (5 male, 4 female). All mice were fed a control diet (LabDiet, Rodent Diet 5001).

### Staining procedure

We used the same brain staining protocol described in [25], which involves the following steps:

1. We used the Non-Survival Surgery procedures approved by the Duke University Institutional Animal Care and Use Committee (IACUC) to sacrifice the mice. We ensured that the sacrifice through exsanguination was carried out humanely with concern for the welfare of our animal subjects. We used a Ketamine (100mg/kg) and Xylazine (10mg/kg) cocktail delivered via the intraperitoneal (IP) route to anesthetize the animal. When the animal was under deep anesthesia (verified by lack of skin and toe pinch reflexes), we performed a conventional transcardiac perfusion fixation with inflow to left ventricle and outflow from right atrium. We then performed perfusion with 0.9% saline containing 0.1% heparin for 5 minutes and switched to 10% formalin flow for another 5 minutes. After perfusion, we cut the head off close to the shoulders, then extracted the brain and stored it in buffered formalin for 3 hours.
2. After the formalin soaking, the brain was dehydrated using a series of ethanol solutions. This dehydration process involved sequential immersion in increasing concentrations of ethanol, including 30%, 50%, 70%, 80%, and 90% ethanol. Immersion lasted 14 hours for 30% ethanol and 12 hours for each of the other four concentrations of ethanol (62 hours total).
3. Next, the brain was stained with a solution of 10% Lugol (containing 10% iodine) and 90% methanol. The staining process took 71 hours. 24 hours after the initial staining, the brain was refreshed with a new 10% Lugol and 90% methanol solution that was used for the remaining 47 hours.
4. Once the staining was completed, the brain was embedded in a 1% agarose gel. The agarose gel was prepared by dissolving 0.1g of agarose gel powder in 10 mL of normal saline (NS). The brain was gently placed in the gel, and the agarose was allowed to solidify. The agarose gel containing the brain was placed in a vial filled with phosphate buffered saline (PBS) for micro-CT scanning.

### CT image acquisition

Our imaging system can acquire data using both a PCD and an EID in sequential acquisitions. The PCD is an XC-Thor, a CdTe-based PCD from Direct Conversion (https://directconversion.com) with two energy thresholds. This detector enables high-resolution imaging (1024 x 256 pixels, 100 μm pixel size) and is appropriate for small animal micro-CT. The detector’s *High Sensitivity with Anti Coincidence* setting was used for all experiments. In this acquisition mode, the PCD counts events surpassing each energy threshold using custom ASIC circuitry and corrects for charge sharing among neighboring pixels by identifying the pixel with the maximum signal as the primary interaction site [40]. X-rays were generated by a L9181-02 39W micro-focus X-ray source (Hamamatsu; tungsten anode; filtration: 0.50 mm beryllium; 16–50 μm spot size). For tomographic scans we used a motorized rotator (Newport, model URS 100BPP) to step the subject through all rotation angles. The subject (vial containing mouse brain) was mounted in a 3D printed cradle during acquisition. To increase the field of view and reduce the effects of ring artifacts via helical scanning, a vertical translator (Thorlabs, MLJ150 high load vertical stage) was used to move the subject during acquisition. An in-house Python script was developed to control motion and image acquisition during tomographic scans [41].

Each brain was scanned consecutively on the PCD and the EID of our ex-vivo micro-CT imaging system. Both the PCD and EID scans were acquired with the same x-ray source settings of 60 kVp and 134 μA and the same helical trajectory of 1200 projections over 1070 degrees rotation and 18 mm vertical translation. For each projection angle, the EID recorded the average of 10 projections with exposure times of 100 ms each, while the PCD recorded the average of 100 projections with exposure times of 80 ms each. Averaging multiple projections with short exposure time results in high SNR without saturating the detectors. Energy thresholds for the PCD were set to 15 and 34 keV. Each EID CT scan took about 1 hour and 46 minutes and each PCD CT scan took about 3 hours and 10 minutes, for a total of 4 hours and 56 minutes of hybrid micro-CT scanning per brain. The EID-based micro-CT scan was acquired to help with corrections for PCD artifacts and to increase the number of available contrasts per brain. We carefully selected the number of projections and exposure time to optimize the tradeoff between total scan time and signal to noise ratio (SNR). Given that we generally had 4 or 5 mouse brains available for scanning per week, using settings that required ~5 hours of scanning per brain allowed us to obtain high SNR images while also scanning all brains within a span of 2 days to avoid variation in brain contrast due to loss of iodine. We note that radiation dose was not a concern because these micro-CT scans were acquired ex vivo on stained brains.

### Artifact correction

The XC-Thor PCD has 8 detector tiles with different spectral response functions. In the image domain, these tile gain differences produce artifacts that look like low frequency concentric rings in the axial view and vertical stripes in the sagittal and coronal views [29]. The EID scans are affected by beam hardening, resulting in cupping artifacts in the image domain.

To reduce concentric artifacts in PCD images and improve background uniformity, we acquired a helical PCD scan of the region of the vial below the brain that contains only PBS using the same settings as our PCD brain scans. We then corrected PCD projections of brains using the multiplicative projection domain water gain correction described by Kim and Baek, with the PBS-only projection used in place of a water phantom [30]. Briefly, this approach involves (1) the creation of an ideal PBS-only vial image, (2) forward projection of this ideal PBS vial, (3) creation of a PBS gain ratio projection through division of the ideal PBS projection by the real one, and (4) multiplication of the sample (e.g. brain) PCD projection by the PBS gain ratio projection. In our implementation, we applied a median filter with neighborhood size of 4×4 pixels to the PBS gain ratio projection to reduce noise amplification in corrected brain images. In addition, we replaced the columns in the PBS gain ratio projection that were outside of the PBS solution with their nearest neighbor column in the PBS to prevent artifacts caused by discrepancies in position of the vial between the brain scan and PBS scan. While there is an additional scan time of 3 hours and 10 minutes to acquire the real PBS projection, the PBS gain ratio projection created from this one scan can be used to correct the PCD projections of all brains because they were all scanned with the same helical trajectory and exposure time.

Beam hardening in the EID projections was corrected using a 2^nd^ degree polynomial function of the log-normalized projection intensity values [29]. The coefficients for this polynomial were calibrated using EID scans of different combinations of acrylic and aluminum plates, with known thickness for each material. The polynomial fitting was done separately for each detector row. The polynomial was evaluated on the log-normalized projections of brains to convert intensity values to acrylic thickness (assumed no aluminum). Then, the corrected projections were rescaled via multiplication with a reference attenuation value for 1 mm of acrylic.

### Image reconstruction

Although multiplicative PBS gain correction greatly reduces tile gain artifacts in PCD projections, some rings remain at the boundaries between tiles in the corrected reconstruction. Therefore, to further reduce ring artifacts, we performed hybrid iterative reconstruction of the brains, with the PBS gain corrected PCD data as the first two energy channels and the beam hardening corrected EID data as the third energy channel. Our reconstructed volumes have an isotropic voxel size of 22 μm, axial slices of size of 880 × 880 voxels, and between 624 and 720 total slices depending on the size of the brain (volume size 19.36 mm × 19.36 mm × 15.84 mm). Previous mouse brain morphometry studies that used diffusion-weighted (DW) MRI scanning required 14 hours of scanning per brain for structural and connectivity information and produced images with isotropic voxel size of 55 μm and matrix size of 368 × 184 × 184 voxels (volume size 20.24 mm × 10.12 mm × 10.12 mm) [37]. Prior studies that acquired T1 and T2 weighted structural MRI scans of mouse brains required 6 hours and 22 minutes of scanning per brain, with T2-weighted images having matrix size of 256 × 256 × 512 voxels and 43 μm isotropic voxel size (volume size 11 mm × 11 mm × 22 mm) [1].

For our iterative reconstructions, we used the split Bregman method with the add-residual-back strategy [42] and rank-sparse kernel regression regularization (RSKR) [34], solving the following optimization problem:

$$\hat{X}=\underset{X}{\arg\;\min}\frac{1}{2}\sum_{e}{\left||RX(e)-Y(e)|\right|}_{2}^{2}+\lambda {\left||X|\right|}_{BTV}.$$ (1)

Thus, we solved iteratively for the vectorized, reconstructed data, the columns of X, for each energy simultaneously (indexed by e). The reconstruction for each energy minimizes the reprojection error (R, system projection matrix) relative to the log-transformed projection data acquired at each energy (the columns of Y). To reduce noise in the reconstructed results, this data fidelity term was minimized subject to the bilateral total variation (BTV) measured within and between energies via RSKR. Each set of 3 energy channel brain data was reconstructed iteratively using 4 total Bregman iterations.

To demonstrate the effects of tile gain and beam hardening corrections prior to hybrid iterative reconstruction, we also generated analytical reconstructions of our brain samples using the weighted filtered backprojection (wFBP) algorithm [43] with a Ram-Lak filter. These reconstructions used the same voxel size and image matrix size as the iterative reconstruction for the same brain. The wFBP reconstructions of PCD and EID data were done separately and then concatenated along the energy dimension to produce the hybrid analytical reconstruction.

### Material decomposition

For image-based material decomposition of the hybrid iterative reconstruction, we used the method of Alvarez and Macovski [44] with H_2_O and Iodine (I) as basis functions:

$$X(e)=C_{H2O}M_{H2O}(e)+C_{I}M_{I}(e).$$ (2)

In this formulation, *M* is a matrix of material sensitivities at each energy computed from a reconstruction of a phantom containing a water vial and vials with known concentrations of I. *C* represents density in g/mL for H_2_O and concentration in water in mg/mL for I. Finally, *X* is the attenuation coefficient of the voxel under consideration at energy *e*. Material decomposition was performed by matrix inversion, solving the following linear system at each voxel:

$$C=XM^{‐1}$$ (3)

Orthogonal subspace projection was used to prevent negative concentrations [34]. Post decomposition, the material maps were assigned to colors and combined and visualized in ImageJ.

### Image quality assessment

To quantify the effect of PBS gain correction, we plot an axial slice of a PCD wFBP reconstruction of a mouse brain sample at both energy thresholds (15 and 34 keV) before and after correction. For each image, we measured the attenuation along a line profile through the center of the vial and report the equation and r^2^ value of the best fit line for this line profile. Using the hybrid iterative reconstruction of the same mouse brain, we measured the maximum and minimum attenuation values along the same line profile and computed percent image uniformity (PIU) in the 15 keV and 34 keV PCD energy channels using the following formula:

$$PIU=100\times \left(1-\frac{\left(max-min\right)}{\left(max+min\right)}\right)$$ (4)

Much like our analysis described above for PBS gain correction, our assessment of beam hardening correction of EID data included displaying an example axial slice of a mouse brain sample, measuring attenuation along a line profile, and plotting the best fit line to this profile both before and after correction. For this brain sample, we measured PIU along the same line profile in the EID channel of the hybrid iterative reconstruction.

To assess spatial resolution, we scanned and reconstructed the QRM MicroCT Bar Pattern Phantom. PCD and EID helical scans for this phantom were acquired with the same settings used for the brains. We generated 3 iterative reconstructions from these scans: PCD only, EID only, and hybrid. For each iterative reconstruction, we computed the modulation transfer function (MTF) by fitting a Gaussian curve to contrast values measured from line profiles through the bar patterns with 3.3, 5, 10, and 16.6 line pairs per mm (lp/mm). For both PCD and hybrid reconstructions, MTF was computed in the PCD 34 keV threshold energy channel. In addition to displaying MTF curves and reporting spatial resolution at 10% MTF, we also display the phantom image slices that were used to compute the MTFs. These quantitative and qualitative assessments help us confirm that the PCD can improve delineation of brain region boundaries.

For additional image quality assessment, we prepared a phantom containing a water vial and vials with four different concentrations of iodine in water: 9.25 mg/mL, 4.625 mg/mL, 2.3125 mg/mL and 1.1563 mg/mL. We scanned this phantom on the PCD and EID with the same settings used for the brains, then performed hybrid iterative reconstruction. For each energy channel in this reconstruction, we extracted attenuation measurements from a region of interest (ROI) in each vial. To assess linearity of the measurements, we converted the attenuation values to Hounsfield Units (HU) and plotted the HU values against the known concentration of iodine. We also plotted the contrast to noise ratio (CNR) at each concentration of iodine, along with a horizontal line representing the upper limit of the Rose criterion for detectability (CNR = 5) [45]. CNR was computed using the following formula:

$$CNR=\frac{\mu_{1}-\mu_{2}}{\sqrt{\frac{\sigma_{1}^{2}+\sigma_{2}^{2}}{2}}}$$ (5)

Where *μ*_1_ and *σ*_1_ are the mean and standard deviation from the iodine vial ROI and *μ*_2_ and *σ*_2_ are the mean and standard deviation from the water vial ROI. For both the QRM phantom and the vials phantom, we applied beam hardening correction to EID data, but did not apply PBS gain correction to PCD data because the phantoms were not immersed in PBS solution.

To estimate noise level in each energy channel before and after iterative reconstruction, we calculated coefficient of variation (CV) using the formula:

$$CV=\frac{\sigma }{\mu }$$ (6)

where *μ* and *σ* are the mean and standard deviation from an ROI.

Since improved contrast between brain regions results in more accurate region segmentations, we generated additional CNR measurements in mouse brains following hybrid iterative reconstruction. These analyses assessed how the CNR changes based on the timing of the scan and how CNR varies between the iodine material map and the three energy channels of the reconstruction. For these calculations, *μ*_1_ and *σ*_1_ were the mean and standard deviation from an ROI in a bright foreground structure of the brain, while *μ*_2_ and *σ*_2_ were the mean and standard deviation from an ROI in a dark background structure of the brain.

### Brain region segmentation

We used the Small Animal Multivariate Brain Analysis (SAMBA) tool [46] to generate segmentations of 330 brain regions (165 on right half, same ones on left half) for each mouse brain. In SAMBA, the user first specifies several images of brains samples as inputs, and then the brains are segmented out of the surrounding image through thresholding and morphological operations. Prior to running this step, it is critical to correct severe image nonuniformities that may adversely affect the quality of the segmentation. After segmentation, the brains are rigidly aligned to an atlas brain with known labels, and a minimum deformation template (MDT) that approximates the average shape of the input brains is generated. Then, brain region labels are transferred to the brains passed through SAMBA via diffeomorphic registration between the atlas brain and the MDT as well as between the MDT and each individual brain. We chose the PCD 34 keV channel of our hybrid iterative reconstructions for use in SAMBA. Initially, we passed images from 15 different mouse brains through SAMBA with a DW MRI atlas that has been used in prior studies [37, 47]. However, due to the difficulty of registration between different imaging modalities, the brain region labels for the PCD MDT had noticeable errors. For this reason, we created a symmetric PCD atlas by manually editing the PCD MDT labels for the left half of the brain in 3D Slicer, mirroring these labels across the center of the brain, and replacing the right half of the PCD MDT brain with a mirror image of its left half. We then confirmed that SAMBA labeling with this new PCD atlas works well when the PCD brain passed into SAMBA has similar contrast to the atlas. Therefore, the resulting PCD-based brain atlas was used for a second SAMBA processing run with the PCD 34 keV threshold channel from hybrid iterative reconstructions of 26 different mouse brains (same 15 used to create PCD CT atlas plus 11 more) as the inputs.

### Statistical analysis

The results of SAMBA labeling of 26 PCD mouse brain images via registration to the PCD atlas were used for statistical analyses. We performed region-based and voxel-based analysis to compare: *APOE22HN* vs *APOE33HN*, *APOE22HN* vs *APOE44HN*, *APOE33HN* vs *APOE44HN*, and male vs female. We used two sided two-sample t tests for comparisons of the size of each brain region as a percent of total brain volume. The p-value for each brain region was adjusted using the Benjamini-Hochberg false discovery rate (FDR) correction, with a 5% threshold [48]. In addition, we compared the total brain volume in mm^3^ by sex and by genotype using analysis of variance (ANOVA) with a significance level of 5%. We used the Statistical Parametric Mapping (SPM) toolbox version 12 [49] with cluster false discovery correction for voxel-based analysis, set at 5%, and an initial forming threshold of 0.05. The inputs to SPM analysis were smoothed versions of the log-transformed Jacobian determinants matrices from diffeomorphic registration of individual brains to the MDT. Smoothing was done with a 3D Gaussian kernel with full width at half maximum (FWHM) of 60 μm.

## Results

### Animal population characteristics

Table 1 shows the number of mice in our study by genotype and sex, while Table 2 shows the age distribution in each group.

**Table 1:** Distribution of mouse brains in our study by genotype and sex.

Genotype	Male	Female	Total
*APOE22HN*	5	3	8
*APOE33HN*	6	3	9
*APOE44HN*	5	4	9
**Total**	16	10	26

**Table 2:** Age distribution of our entire mouse population and within genotype and sex groups.

Group (# of mice)	Age in Days (mean ± std dev)
*APOE22HN* (8)	439 ± 43
*APOE33HN* (9)	422 ± 44
*APOE44HN* (9)	380 ± 11
Male (16)	405 ± 46
Female (10)	425 ± 36
**All Mice (26)**	**413 ± 43**

### CT corrections

**Fig 1:**
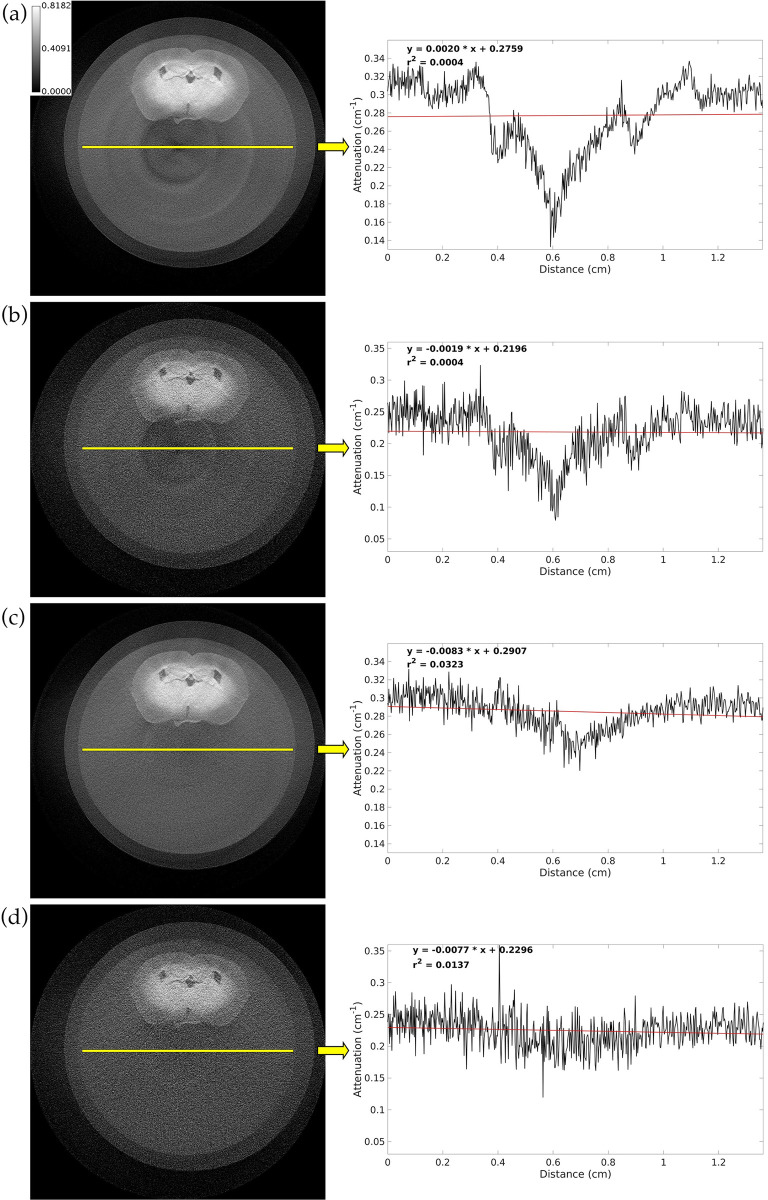
Axial slice from PCD CT analytical reconstruction of a vial with mouse brain before and after tile gain correction using the PBS gain ratio projection. (a) Uncorrected 15 keV image. (b) Uncorrected 34 keV image. (c) Corrected 15 keV image. (d) Corrected 34 keV image. The plot to the right of each image shows the intensity along a line profile through the center of the vial (black curve), the best fit line through this profile (red line), and the equation and r^2^ value for the best fit line. The calibration bar shows the display settings for all images in units of cm^-1^.

Fig 1 shows a slice in axial orientation from the PCD CT analytical reconstruction of a of a mouse brain at both the 15 keV and 34 keV energy thresholds before and after tile gain correction. If the tile gain correction were perfect, we would expect the line profile to have fluctuations in attenuation due to random noise but still maintain a horizontal shape overall. The r^2^ value for the best fit line is small in all 4 images due to high noise level. However, the corrected images have much larger r^2^ values because their line profiles do not have the drastic fluctuations between sharp increasing and decreasing trends that are seen in the line profiles from the uncorrected images. The line profile of the corrected 15 keV image does still have a slight decrease in attenuation at the center of the vial that is likely caused by beam hardening, but the line profile of the corrected 34 keV image does not have this issue. Following hybrid iterative reconstruction of this brain, we determined that the PIU along the line profile is 84.6210% in the 15 keV image and 84.7036% in the 34 keV image.

**Fig 2:**
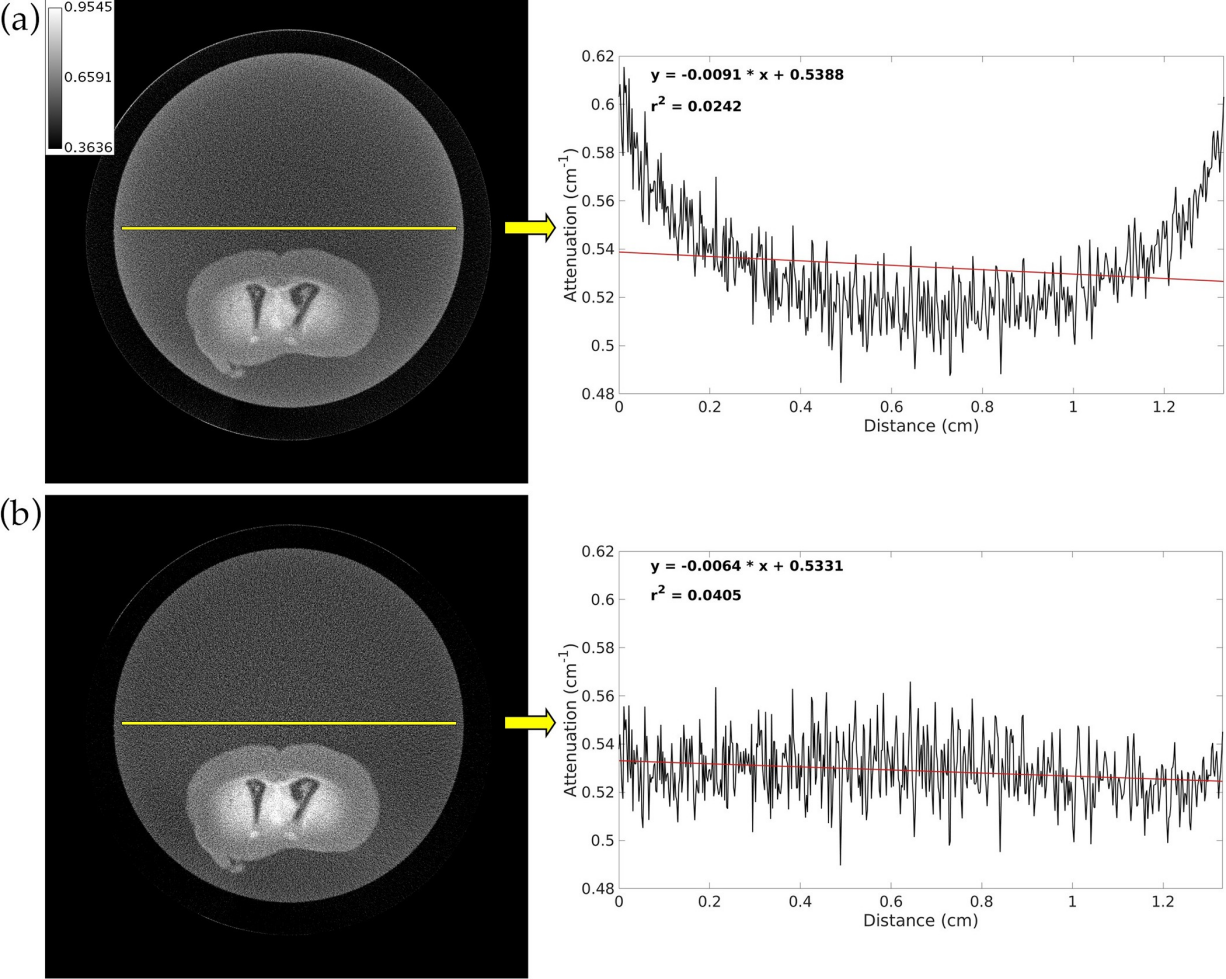
Axial slice from EID CT analytical reconstruction of a vial with mouse brain before and after beam hardening correction. (a) Uncorrected image. (b) Corrected image. The plot to the right of each image shows the intensity along a line profile through the center of the vial (black curve), the best fit line through this profile (red line), and the equation and r^2^ value for the best fit line. The calibration bar shows display settings for both images in units of cm^-1^.

Fig 2 shows an EID CT analytical reconstruction of a mouse brain before and after beam hardening correction. We expect the line profile from the uncorrected EID image to have a parabolic cupping artifact pattern in which the attenuation at the center of the vial is much lower than the attenuation at the edge of the vial. After correction, we expect the line profile to maintain an overall horizontal shape despite fluctuations due to random noise. The results in line profiles in Fig 2 are consistent with this expectation. The r^2^ value of the best fit line for the line profile from the uncorrected image is very small due to both the high noise level and the parabolic shape of the line profile that is typical of cupping artifacts. Although the corrected image still has a small r^2^ value due to high noise level, it no longer has a parabolic shape in its line profile, resulting in a ~1.7-fold increase in the r^2^ value. Following hybrid iterative reconstruction of this brain, we determined that the PIU along the line profile is 87.9414% in the EID image.

### Analysis of hybrid reconstructions

**Fig 3:**
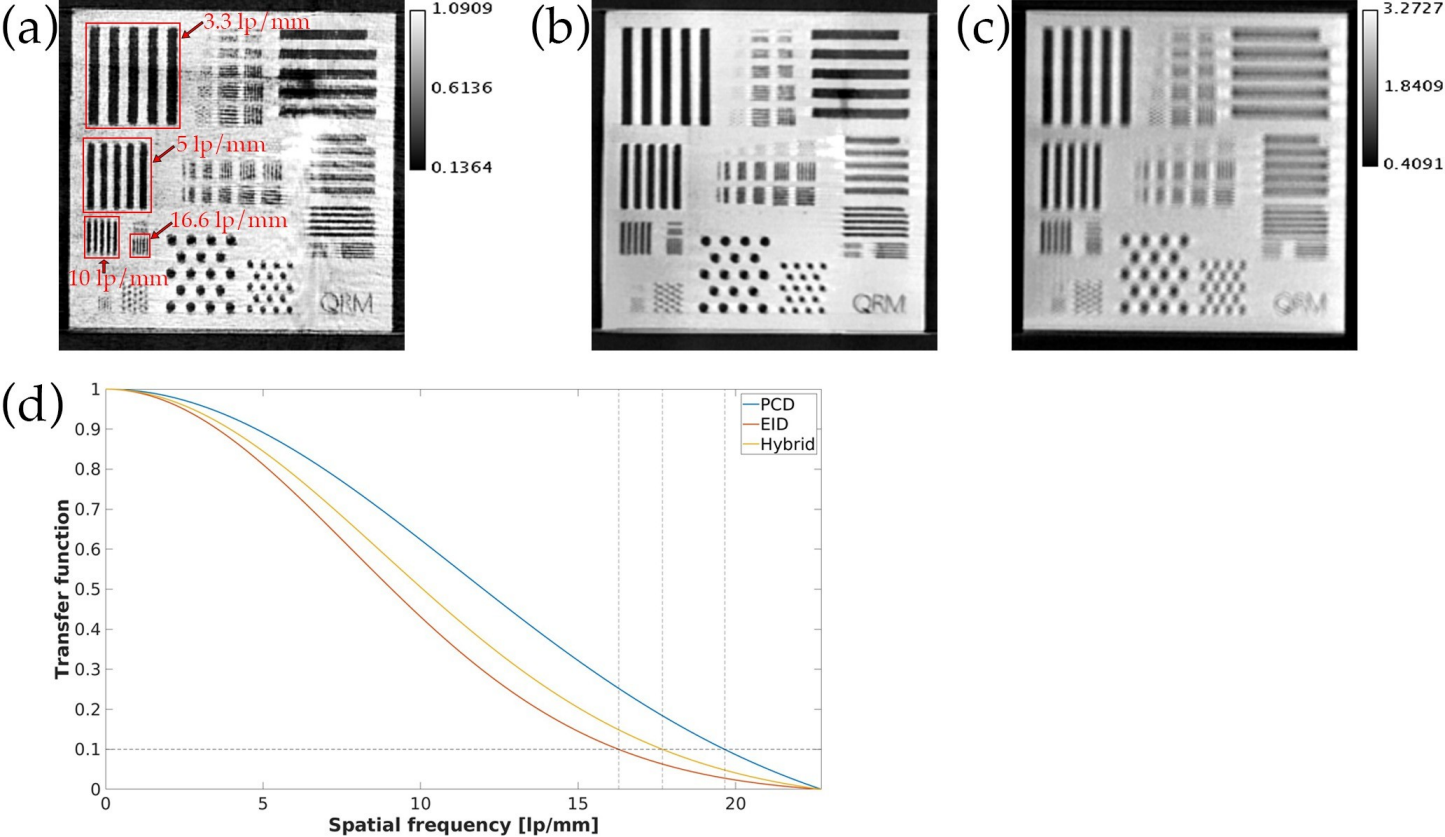
Spatial resolution assessment using QRM phantom. (a) 34 keV image from PCD iterative reconstruction. (b) 34 keV image from hybrid iterative reconstruction. (c) Image from EID iterative reconstruction. (d) MTFs derived from bar patterns in (a,b,c). The red rectangles in (a) highlight the four different types of bar patterns used to compute the MTF for each energy.

Fig 3 shows the reconstructed images and MTFs from scanning the QRM phantom. The PCD 34 keV image has a spatial resolution of 19.7 lp/mm at 10% MTF in a PCD iterative reconstruction and 17.7 lp/mm at 10% MTF in a hybrid iterative reconstruction. The spatial resolution at 10% MTF is 16.3 lp/mm in an iterative reconstruction using only EID. When looking at the bar patterns on the reconstructed images, it is clear that the EID image in Fig 3C is severely compromised by spatial blurring and partial volume effects, while the PCD image in Fig 3A achieves high spatial resolution but is also affected by stripes from tile gain artifacts. Fig 3B shows that the hybrid reconstruction results in PCD images that have minimal artifacts and superior spatial resolution to EID images despite some spatial blurring.

**Fig 4:**
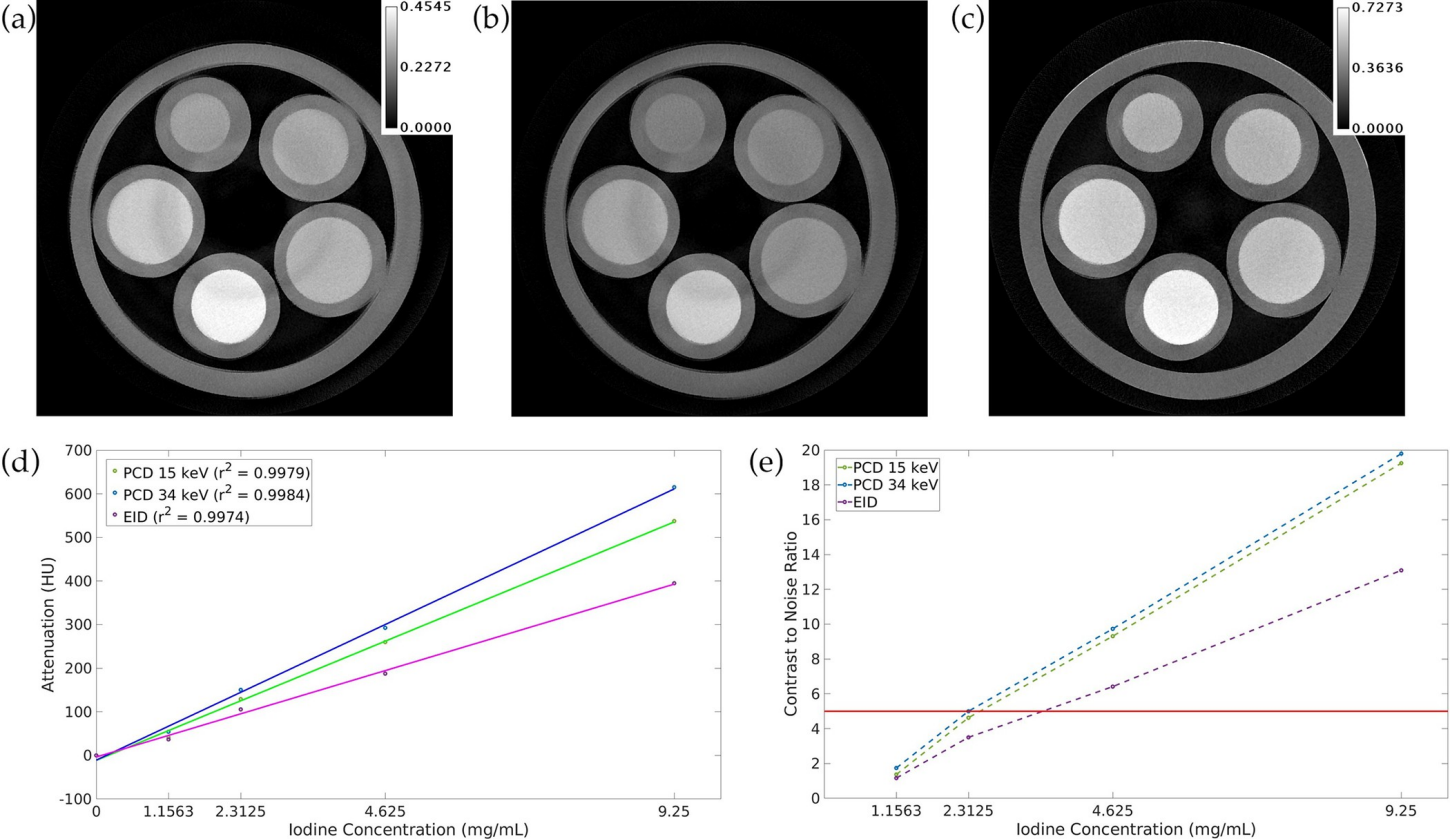
Linearity and CNR assessment using phantom with water and iodine vials. (a) PCD 15 keV image. (b) PCD 34 keV image. (c) EID image. (d) Linearity plots for all 3 energy channels. (e) CNR plots for all 3 energy channels. The horizontal red line in (e) represents the upper limit of the Rose criterion for detectability (CNR = 5) [45].

Fig 4 shows an axial slice from the hybrid iterative reconstruction of our phantom with water and iodine vials as well as the resulting linearity and CNR plots. All three energies show high linearity, with r^2^ values above 0.997. At each iodine concentration, the PCD 34 keV image has the highest CNR, followed by the PCD 15 keV image and then the EID image. In the PCD 34 keV image, 3 out of 4 iodine vials have CNR above the upper limit of the Rose criterion. Note that the iodine phantom images had no PBS-based tile corrections since they were not immersed in PBS solution; thus some concentric ring artifacts are still visible.

**Fig 5:**
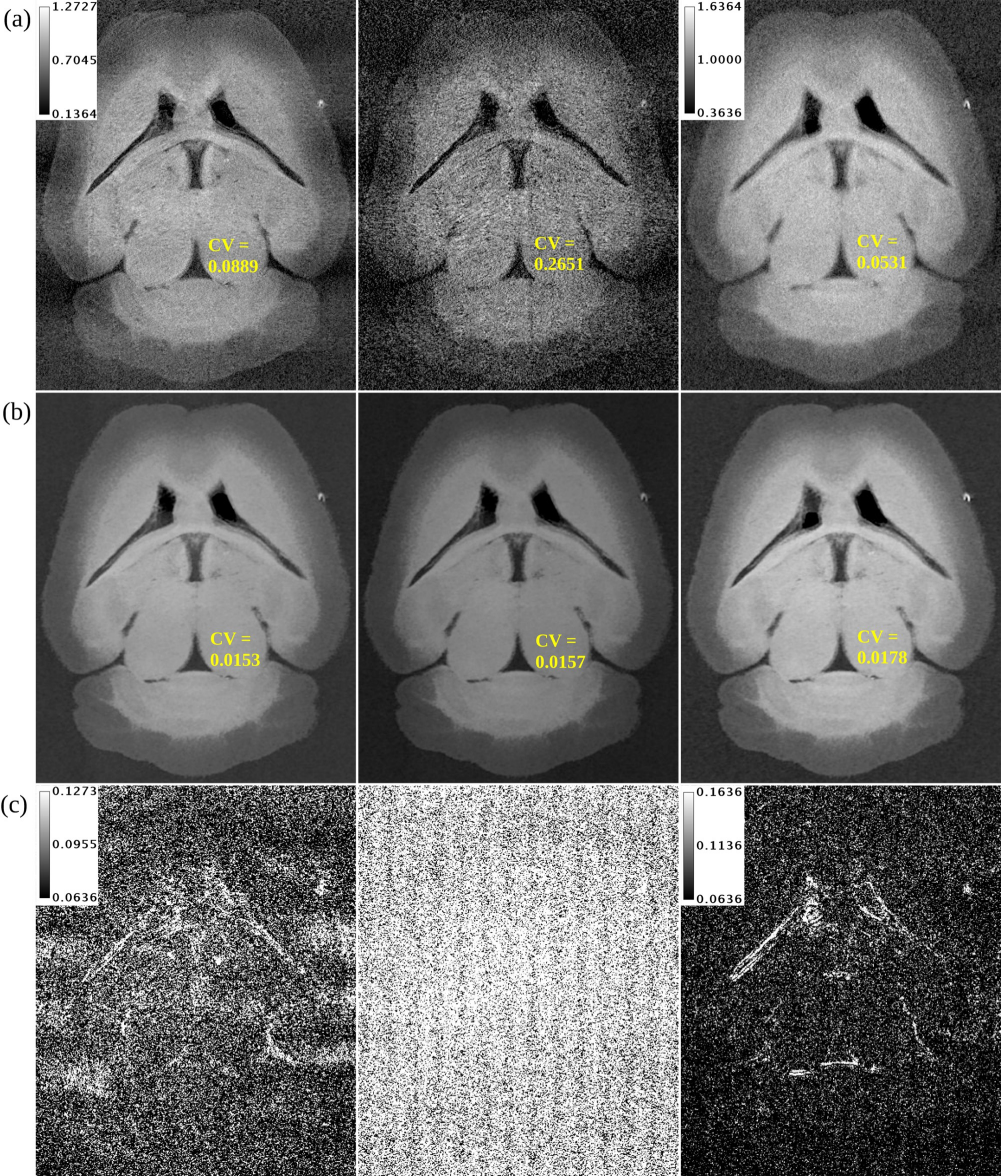
Coronal slice from hybrid reconstruction of a mouse brain. (a) PCD 15 keV threshold (left), PCD 34 keV threshold (center), and EID (right) channels of hybrid analytical reconstruction of a mouse brain. (b) Hybrid iterative reconstruction of the same mouse brain. (c) Absolute difference between (a) and (b). The calibration bars show the display settings for PCD images, PCD absolute difference maps, EID images, and EID absolute difference map in units of cm^-1^. CV measurements from the inferior colliculus show substantial reduction in noise following hybrid iterative reconstruction (a vs. b).

Fig 5A shows the hybrid analytical reconstruction of a mouse brain. Qualitative comparison between images from the two detectors supports the finding of higher spatial resolution in PCD images. This is likely because only the PCD detects x-ray photons without a scintillator, as discussed earlier [27]. Following joint hybrid reconstruction of the two imaging chains as seen in Fig 5B, the CV (measured from ROI in inferior colliculus) in all energy channels decreases dramatically and the spatial resolution in the EID channel improves. Due to imperfection of the PBS gain correction, the PCD images in Fig 5A still have some faint stripe artifacts that are not present in the EID image. The iterative reconstruction algorithm attempts to resolve this discrepancy during joint regularization of the PCD and EID channels. As a result, the absolute difference images in Fig 5C have a vertical stripe pattern.

**Fig 6:**
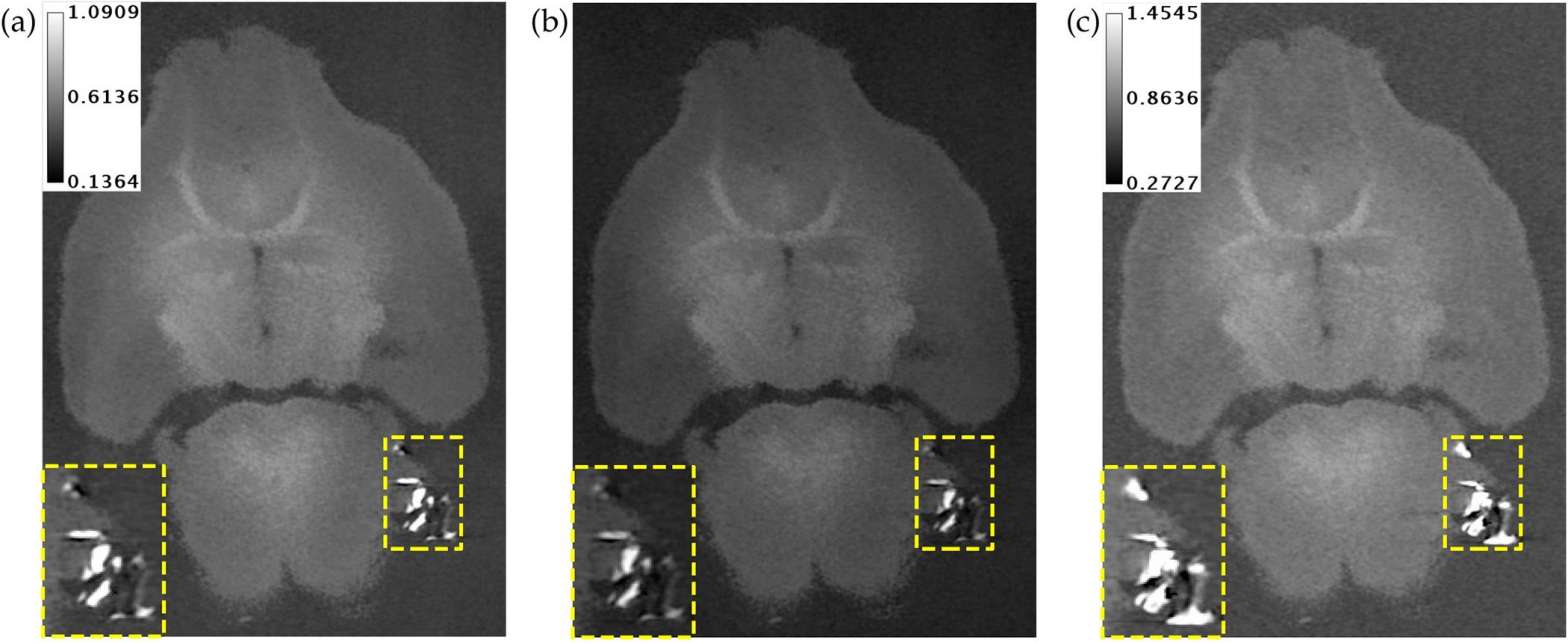
Coronal slice from hybrid iterative reconstruction of a mouse brain with small pieces of skull attached. (a) PCD 15 keV threshold. (b) PCD 34 keV threshold. (c) EID. Both PCD channels show much less calcium blooming than the EID channel. The calibration bars show the display settings for PCD and EID images in units of cm^-1^.

Fig 6 shows a hybrid iterative reconstruction of a mouse brain with pieces of the skull attached due to an imperfect preparation procedure. As expected, the 34 keV threshold PCD channel shows much less calcium blooming of the skull pieces than the EID channel. However, even the 15 keV threshold PCD channel has less calcium blooming than the EID. Reduction of this artifact prevents brain regions at the exterior from being obscured by fragments of bone.

**Fig 7:**
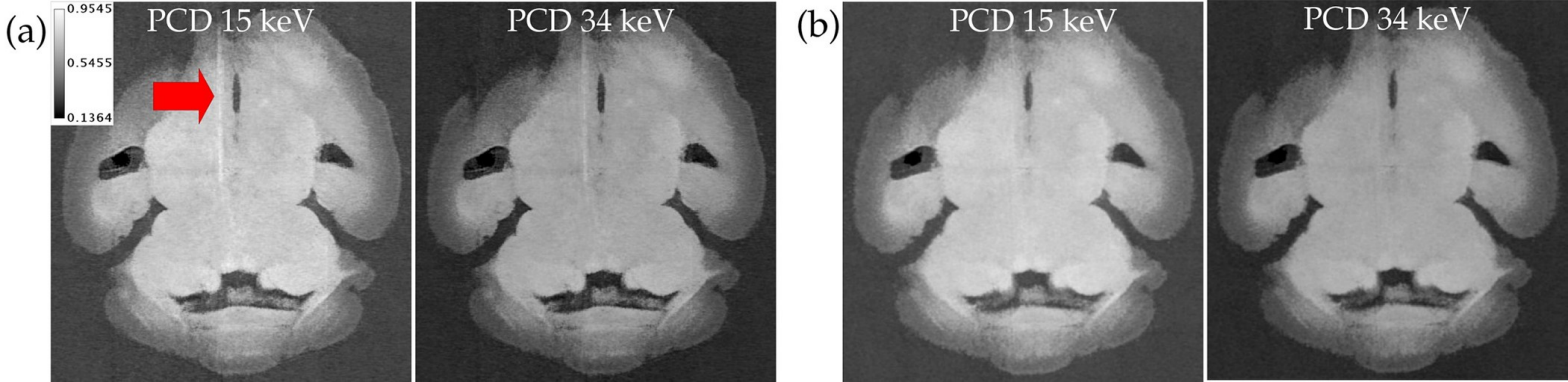
PCD only iterative reconstruction versus hybrid iterative reconstruction of same mouse brain in a coronal slice. (a) PCD 15 keV threshold and PCD 34 keV threshold from iterative reconstruction of PCD only. (b) PCD 15 keV threshold and PCD 34 keV threshold from hybrid (PCD & EID) iterative reconstruction. The red arrow in (a) shows an artifact that is caused by errors in the PBS gain correction at the boundaries between neighboring detector tiles. This artifact is dramatically reduced in the hybrid reconstruction in (b). The calibration bar in (a) shows the display setting for all images in units of cm^-1^.

Fig 7 compares a PCD only iterative reconstruction of a mouse brain to the PCD channels of a hybrid iterative reconstruction of the same brain. Despite the multiplicative PBS gain correction, the PCD images still have some artifacts on the brain near the boundaries between neighboring detector tiles. Including EID data in the iterative reconstruction reduces these artifacts.

**Fig 8:**
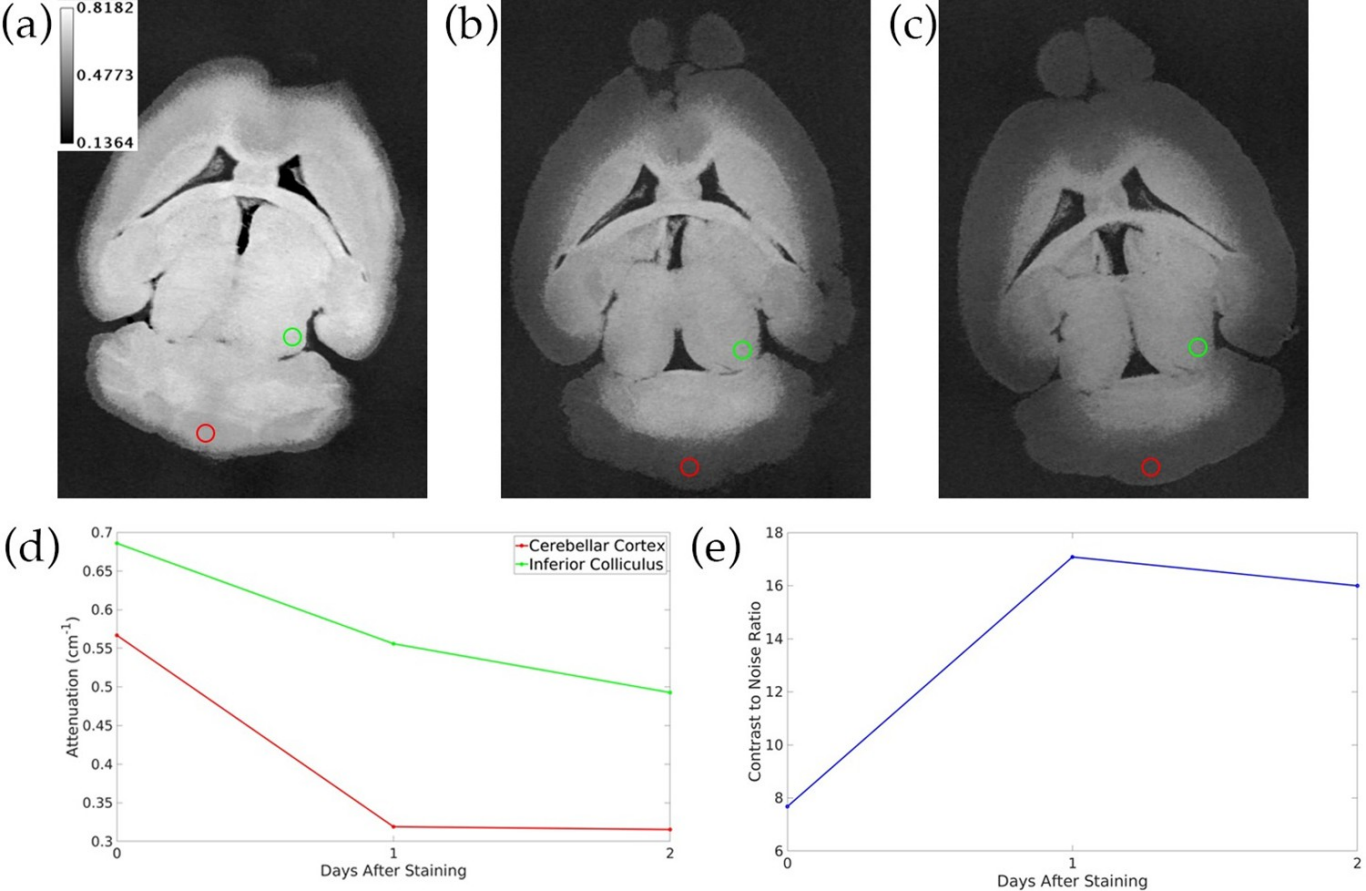
Coronal view of mouse brains scanned at different time intervals after preparation. (a) Brain scanned immediately after completion of staining procedure. (b) Brain scanned 1 day later. (c) Brain scanned 2 days later. Mean attenuation in inferior colliculus and cerebellar cortex (d) and CNR (e) as functions of number of days after staining that the brain was scanned. CNR calculations treated inferior colliculus as the foreground and cerebellar cortex as the background. The green and red circles in (a-c) indicate the inferior colliculus and cerebellar cortex ROIs used for the quantitative analysis in (d,e). All images shown are the 34 keV threshold PCD image from hybrid iterative reconstruction. The calibration bar shows the display settings for all images in units of cm^-1^.

Fig 8 shows the PCD 34 keV threshold channel of a hybrid iterative reconstruction for 3 different brains, each scanned at different time intervals after completion of the staining procedure, as well as quantitative measurements taken from ROIs in the inferior colliculus and cerebellar cortex. Attenuation values from these two brain regions suggest that interior brain regions (e.g. part of the inferior colliculus) slowly lose contrast agent at a roughly constant rate, while exterior regions such as the cerebellar cortex lose all contrast agent within a day. For the 15 brains that contributed to the creation of the PCD atlas, we chose to do our scans 1 or 2 days after staining due to the improved contrast between brain regions. Therefore, we also scanned the remaining 11 brains 1 or 2 days after staining to prevent SAMBA labeling errors due to discrepancies in contrast. We have observed that the brains no longer retain any contrast agent after about 7 days.

**Fig 9:**
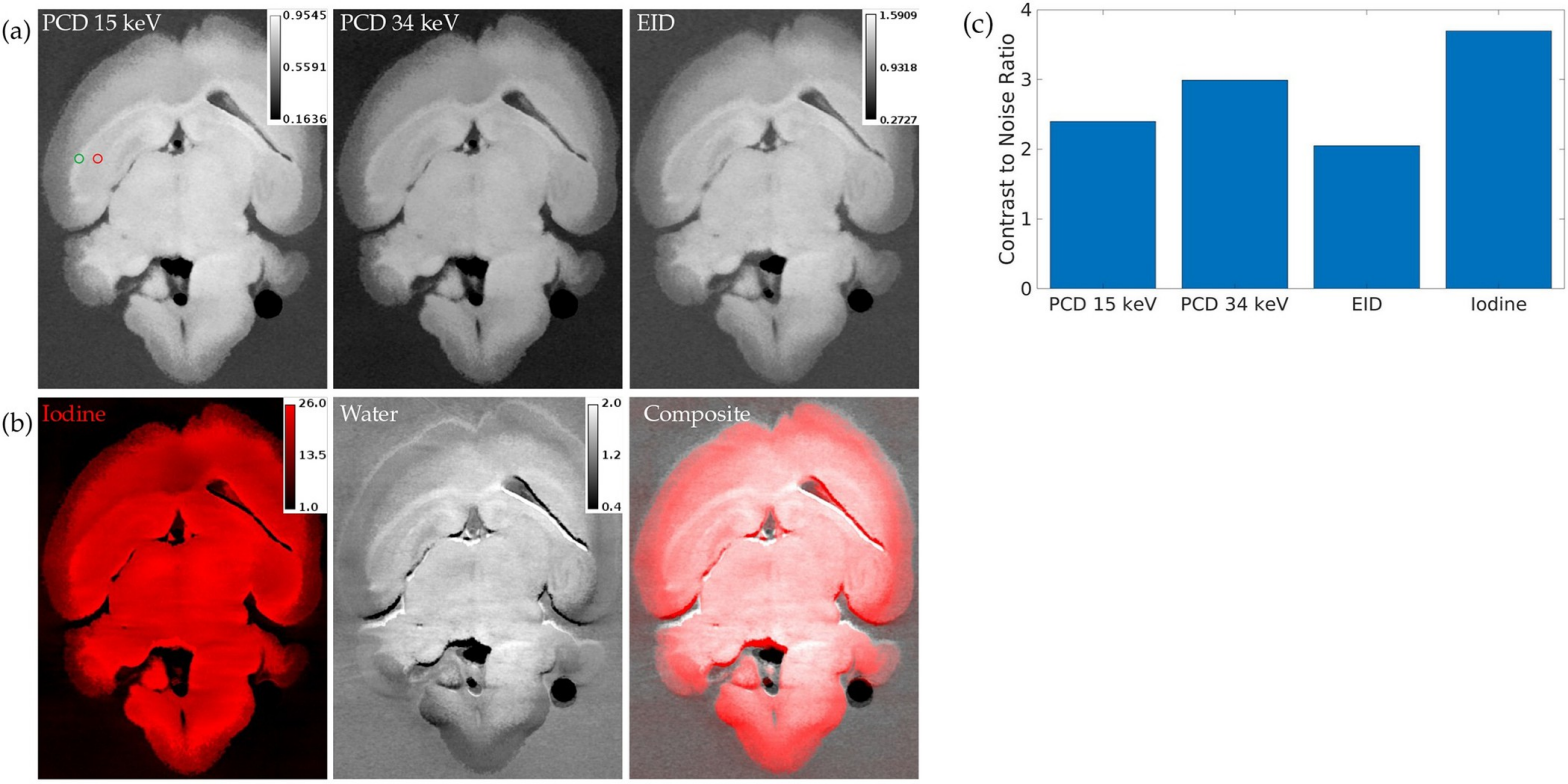
Coronal slice of hybrid iterative reconstruction and material decomposition of a mouse brain. (a) PCD 15 keV threshold, PCD 34 keV threshold, and EID channels of hybrid iterative reconstruction. (b) Iodine, water, and composite maps from material decomposition of hybrid iterative reconstruction. (c) CNR from ROI measurements. The green and red circles in the PCD 15 keV threshold image show the ROIs in the corpus callosum (foreground) and hippocampus (background) that were used to calculate CNR in each image. The calibration bars in (a) show the display settings for the PCD and EID images in units of cm^-1^, while the iodine calibration bar indicates concentration in water in mg/mL and the Water calibration bar indicates density in g/mL.

Fig 9 shows a hybrid iterative reconstruction of a mouse brain as well as the iodine/water material decomposition of this reconstruction. CNR measurements between the corpus callosum and hippocampus show that the iodine map has the highest CNR. Both PCD thresholds provide higher CNR than the EID image. We have selected the PCD 34 keV threshold image for use in SAMBA processing because it consistently produces higher CNR than the PCD 15 keV and EID channels of our hybrid iterative reconstructions.

### SAMBA region labeling performance

**Fig 10:**
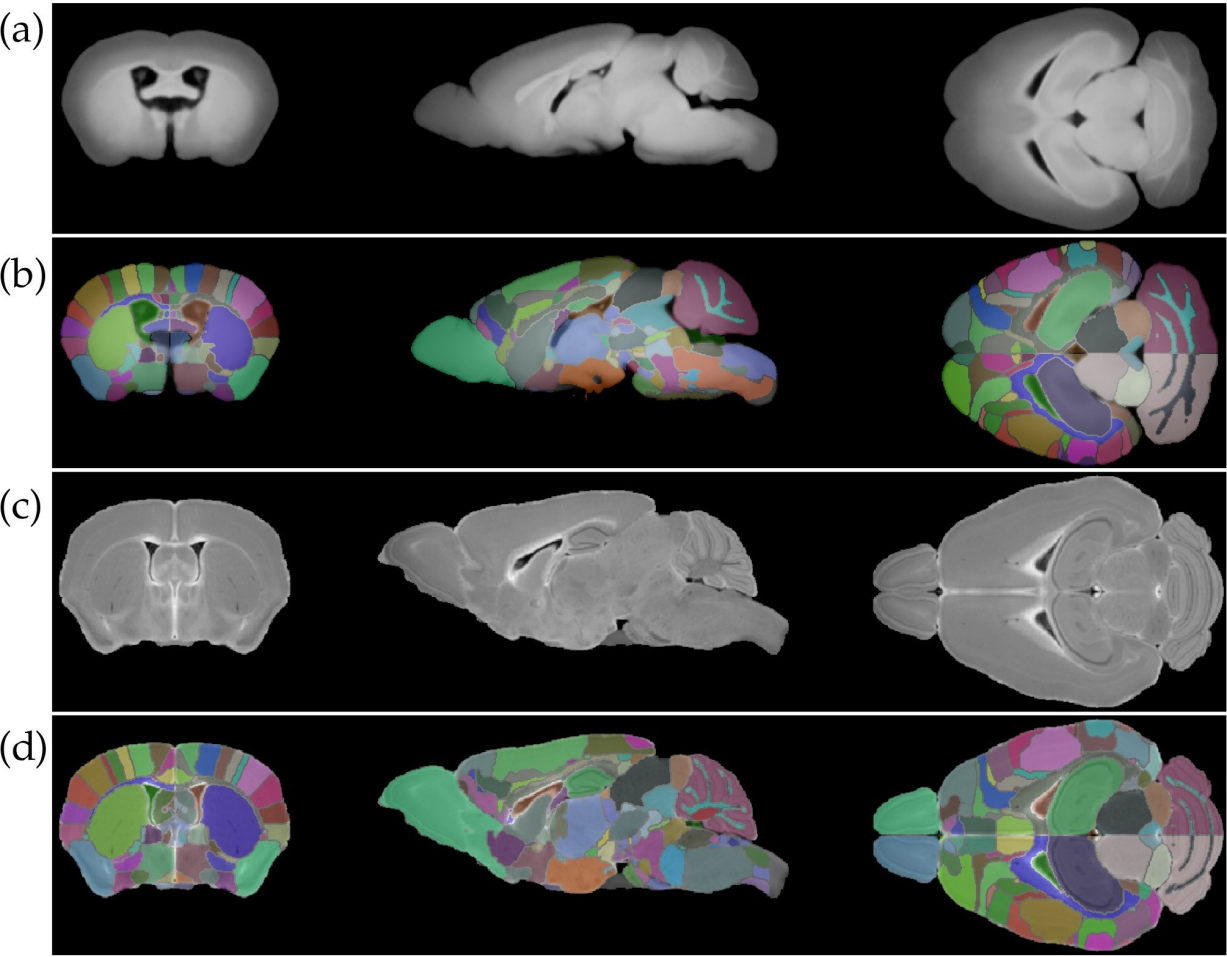
Comparison of atlas brains from PCD CT and DW MRI. (a) PCD 34 keV threshold atlas without labels. (b) PCD 34 keV threshold atlas with labels. (c) DW MRI atlas without labels (d) DW MRI atlas with labels.

Fig 10 shows the PCD atlas with 34 keV energy and the DW MRI atlas, while columns 2 and 3 of Table 3 provide a quantitative comparison of the two atlases in significant brain regions. We acknowledge that the atlases have a large difference in total volume due to different brain staining and fixation procedures for DW MRI and PCD CT. In addition, their shapes are slightly different because the MDTs used to create the DW MRI atlas and PCD CT atlas were not computed with brains from the same mouse strains. Despite these factors, the segmentations of large structures look very similar qualitatively in the two atlases, and each of the brain regions in Table 3 occupies a similar proportion of total brain volume in both atlases.

**Table 3:** Comparison of brain regions and total brain volume between DW MRI atlas, PCD CT atlas, and PCD CT image of *APOE44HN* female mouse brain from Fig 11. These 5 brain regions were chosen because they are large and easily identified in both atlases. For each region, its volume in the left hemisphere is reported as a percent of the total brain volume (last row).

Brain Region	Volume in DW MRI Atlas	Volume in PCD CT Atlas	Volume in PCD CT image of an *APOE44HN* Female Mouse Brain
Hippocampus	3.0734	2.3803	2.5703
Piriform Cortex	5.4556	4.8994	4.0685
Striatum	2.6279	2.3807	2.6560
Cerebellar Cortex	4.4881	5.0326	5.2223
Ventricle	0.3077	0.8307	0.7688
Total Brain Volume (mm^3^)	496.8673	244.9554	187.3734

**Fig 11:**
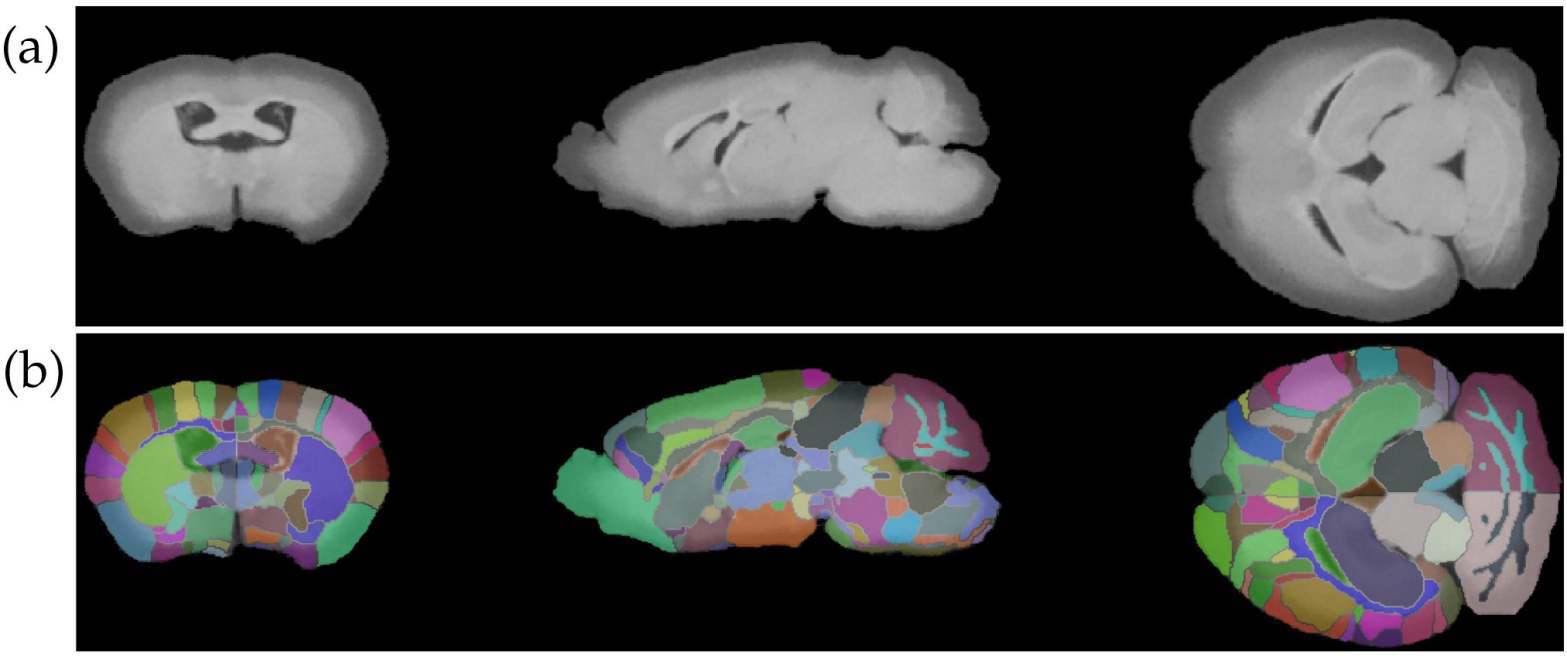
PCD 34 keV image of an *APOE44HN* female mouse brain with labels from SAMBA processing with the PCD CT atlas. (a) Coronal, sagittal, and axial views of brain. (b) Same brain with overlayed labels from SAMBA.

Fig 11 shows the region labeling results from SAMBA registration with PCD CT atlas on a PCD 34 keV image of a brain from a female mouse of the *APOE44HN* genotype associated with high AD risk [37]. Column 4 of Table 3 shows quantitative information for this brain in significant regions.

Although this brain’s volume is 23.5% smaller than the PCD CT atlas, its significant regions occupy a similar proportion of total brain volume to the same regions in the PCD atlas, indicating successful transfer of labels by SAMBA.

### Statistical analysis

**Fig 12:**
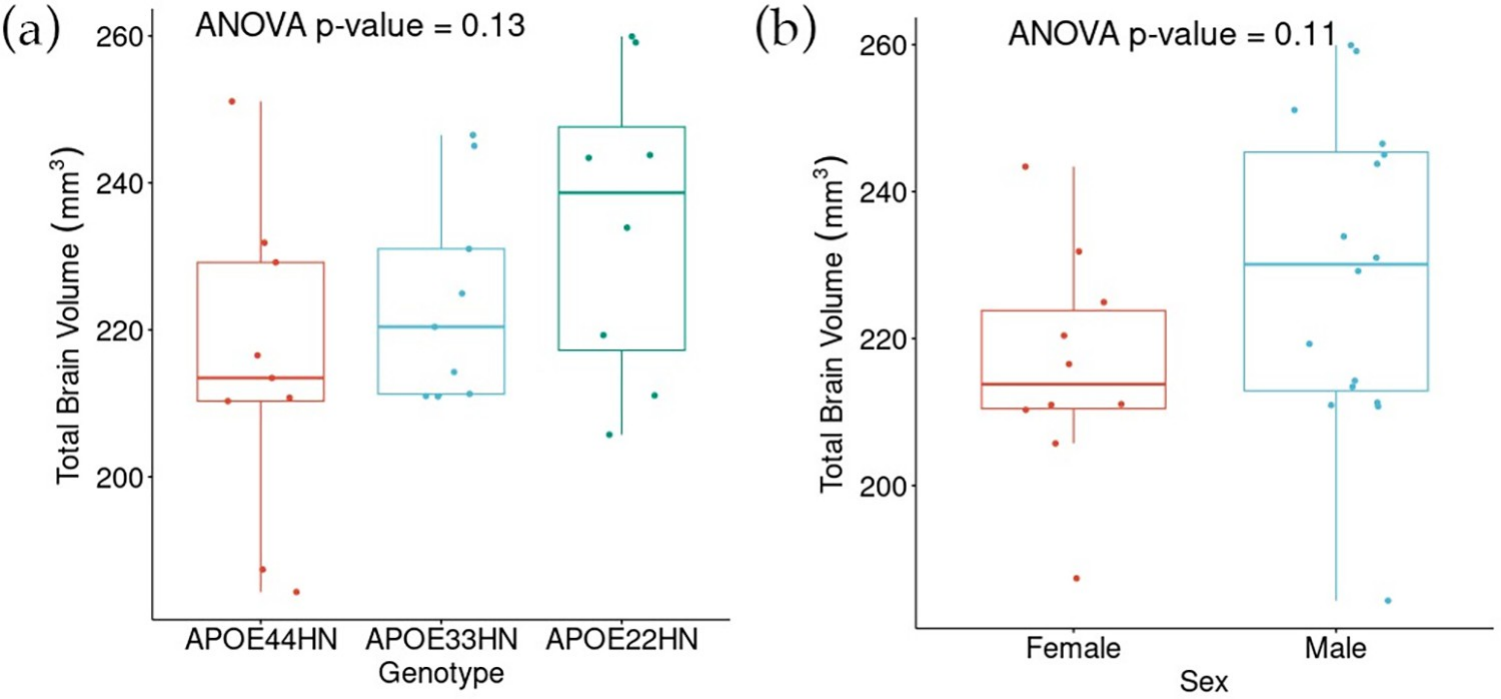
Total brain volume comparison. (a) By genotype. (b) By sex. The p-values from ANOVA across all groups are overlayed on the boxplots.

Fig 12 shows comparisons of total brain volume by genotype and sex using ANOVA. Though the differences weren’t statistically significant, some patterns emerged: *APOE44HN* mice exhibited the smallest median brain volumes, suggestive of atrophy, while *APOE22HN* mice had the largest. Furthermore, on average, male brain volumes appeared larger than female volumes. This observation aligns with existing knowledge; much like in humans, male mice generally possess greater absolute brain sizes than their female counterparts, often reflecting overall body size disparities [50].

In the region-based analysis, significant differences between male and female brain regions emerged after adjusting for multiple comparisons using the Benjamini-Hochberg FDR correction. 26 such regions, which displayed p-values under the 5% significance threshold, are detailed in Table 4.

**Table 4:** Differentiated brain regions in male vs. female mice comparison. The table displays brain regions with statistically significant size disparities between male and female mice across all genotypes. The provided mean and standard deviation values represent the size distribution of these regions as a percentage of the total brain volume. The column "pFDR" shows p-values adjusted for the False Discovery Rate; values under 0.05 indicate significance after adjustment. "Lower Bound of 95% CI" and "Upper Bound of 95% CI" delineate the range within which the true difference is likely to lie, offering an estimate of uncertainty surrounding the mean difference. The "t" column indicates the magnitude of the difference adjusted for within-group variability, stemming from the t-test. Cohen’s d represents the effect size, with larger values indicating more pronounced differences between groups. Lastly, "Difference (%)" highlights the percentage difference between male and female brain regions, with positive values suggesting the region occupies a larger proportion of total brain volume in females and negative values indicating the opposite.

structure	Female (mean ± SD)	Male (mean ± SD)	pFDR	Lower Bound of 95% CI	Upper Bound of 95% CI	t	Cohen d	Difference (%)
**Left Cingulate Cortex, Area 24a prime**	0.0382 ± 0.0076	0.0274 ± 0.0072	0.0435	0.0048	0.0169	3.6767	1.4821	39.6557
**Left Cingulate Cortex, Area 24b**	0.1570 ± 0.0355	0.1209 ± 0.0212	0.0474	0.0133	0.0589	3.2636	1.3156	29.8490
**Left Frontal Association Cortex**	0.7325 ± 0.1570	0.9963 ± 0.1914	0.0435	-0.4129	-0.1146	-3.6500	-1.4714	-26.4741
**Left Lateral Parietal Association Cortex**	0.0264 ± 0.0028	0.0297 ± 0.0023	0.0469	-0.0054	-0.0012	-3.2921	-1.3271	-11.1158
**Left Parietal Cortex, Posterior Area, Rostral Part**	0.0079 ± 0.0008	0.0092 ± 0.0008	0.0435	-0.0020	-0.0006	-3.9819	-1.6052	-14.2450
**Left Primary Somatosensory Cortex, Trunk Region**	0.0735 ± 0.0064	0.0815 ± 0.0050	0.0439	-0.0127	-0.0034	-3.5943	-1.4489	-9.8705
**Left Primary Visual Cortex**	0.0647 ± 0.0060	0.0718 ± 0.0047	0.0469	-0.0114	-0.0027	-3.3549	-1.3524	-9.8469
**Left Secondary Visual Cortex, Mediolateral Area**	0.1340 ± 0.0157	0.1523 ± 0.0126	0.0469	-0.0299	-0.0069	-3.2920	-1.3270	-12.0525
**Left Ventral Orbital Cortex**	0.1966 ± 0.0587	0.1365 ± 0.0309	0.0468	0.0240	0.0963	3.4339	1.3843	44.0746
**Left Globus Pallidus**	0.1914 ± 0.0261	0.1615 ± 0.0184	0.0468	0.0120	0.0479	3.4418	1.3874	18.5474
**Left Striatum**	2.4807 ± 0.2623	2.1423 ± 0.2044	0.0435	0.1489	0.5280	3.6849	1.4854	15.7966
**Left Cingulate Cortex, Area 25**	0.0100 ± 0.0014	0.0080 ± 0.0012	0.0435	0.0009	0.0030	3.7012	1.4920	24.1861
**Left Intermediate Reticular Nucleus**	0.1986 ± 0.0320	0.1552 ± 0.0323	0.0469	0.0166	0.0701	3.3438	1.3479	27.9404
**Right Primary Visual Cortex, Monocular Area**	0.4876 ± 0.0507	0.5442 ± 0.0392	0.0488	-0.0932	-0.0201	-3.2009	-1.2903	-10.4070
**Right Dorsal Intermediate Entorhinal Cortex**	0.1890± 0.0236	0.2296 ± 0.0350	0.0488	-0.0666	-0.0146	-3.2220	-1.2988	-17.6723
**Right Perirhinal Cortex**	0.2269 ± 0.0254	0.2570 ± 0.0218	0.0488	-0.0494	-0.0108	-3.2158	-1.2963	-11.7110
**Right Amygdalopiriform Transition Area**	0.0540 ± 0.0085	0.0656 ± 0.0071	0.0435	-0.0179	-0.0051	-3.7233	-1.5009	-17.5643
**Right Deep Mesencephalic Nuclei**	0.1876 ± 0.0183	0.2147 ± 0.0208	0.0469	-0.0436	-0.0105	-3.3673	-1.3574	-12.6009
**Right Midbrain Reticular Nucleus**	0.5093 ± 0.0313	0.5673 ± 0.0498	0.0469	-0.0945	-0.0216	-3.2856	-1.3245	-10.2285
**Right Pedunculotegmental, Medial Paralemniscial, and Supratrigemnial Nuclei**	0.0544 ± 0.0042	0.0473 ± 0.0037	0.0435	0.0038	0.0102	4.5178	1.8212	14.8482
**Right Motor Root of Trigeminal Nerve**	0.0097 ± 0.0019	0.0077 ± 0.0010	0.0468	0.0008	0.0032	3.4866	1.4055	26.0821
**Right Trigeminal Motor Nucleus**	0.0258 ± 0.0059	0.0194 ± 0.0037	0.0468	0.0025	0.0102	3.4171	1.3775	32.8332
**Right Pontine Reticular Nucleus Oral**	0.7217 ± 0.0457	0.6604 ± 0.0393	0.0435	0.0265	0.0961	3.6357	1.4656	9.2833
**Right Pontine Reticular Nucleus Caudal**	0.2928 ± 0.0249	0.2499 ± 0.0267	0.0435	0.0212	0.0645	4.0860	1.6471	17.1516
**Right Cingulate Cortex, Area 25**	0.0091 ± 0.0011	0.0075 ± 0.0012	0.0468	0.0007	0.0025	3.5295	1.4228	21.5360
**Right Intermediate Reticular Nucleus**	0.2070 ± 0.0359	0.1636 ± 0.0204	0.0435	0.0207	0.0661	3.9442	1.5899	26.5222

Although we did not find significant differences by genotype in region-based analysis, we have provided S1 Table to show the mean and standard deviation of total brain volume (mm^3^) and the size of each of our 330 brain regions (% of total brain volume) for each genotype.

**Fig 13:**
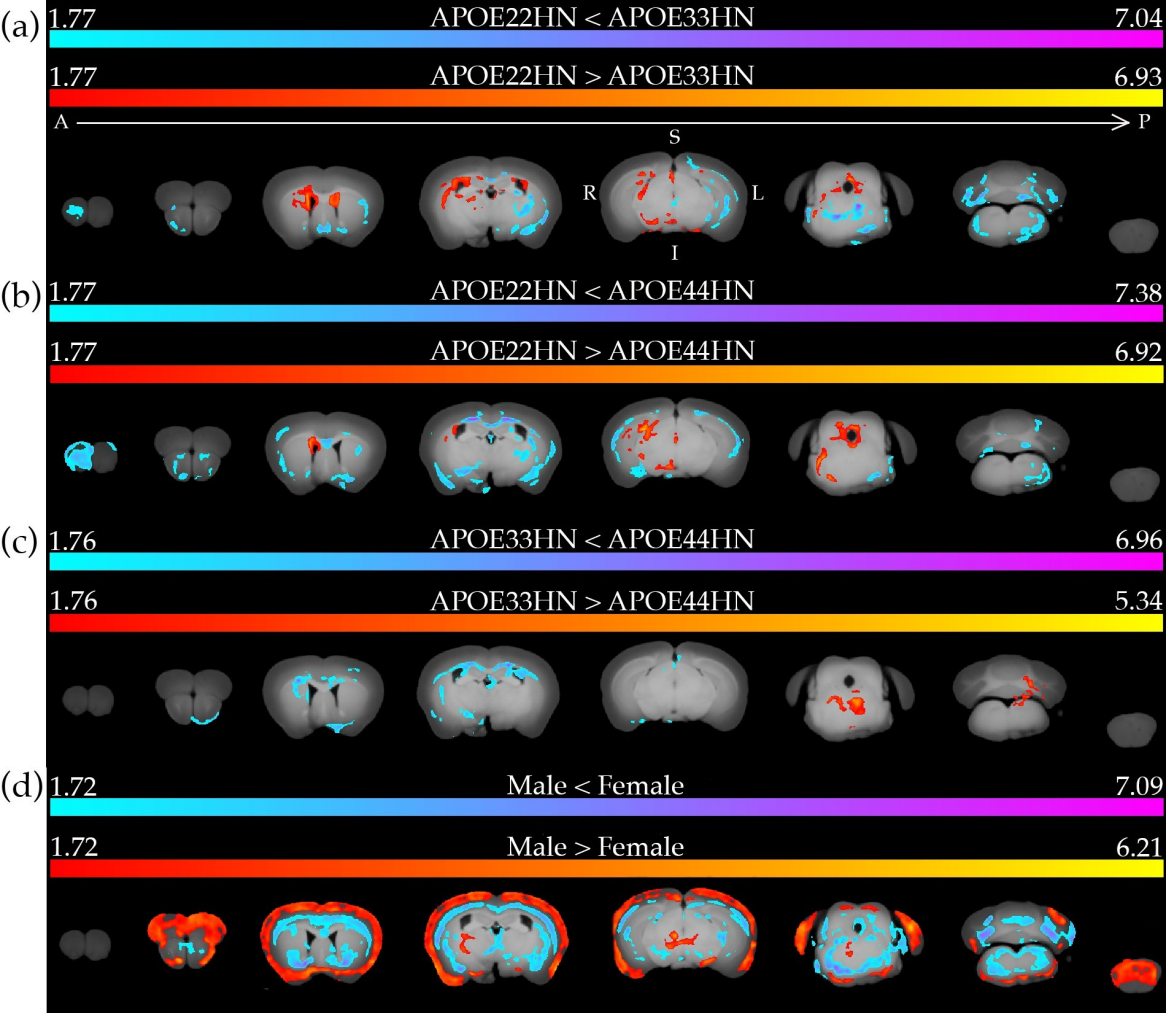
Results of voxel-based analysis using SPM. (a) Positive and negative effect of *APOE22HN* vs *APOE33HN*. (b) Positive and negative effect of *APOE22HN* vs *APOE44HN*. (c) Positive and negative effect of *APOE33HN* vs *APOE44HN*. (d) Positive and negative effect of Male vs Female. Labels in (a) indicate the left/right (L/R), superior/inferior (S/I), and anterior/posterior (A/P) directions.

Fig 13 shows the significant clusters from voxel-based analysis with SPM. When comparing *APOE22HN* versus *APOE33HN*, we noted *APOE22HN* mice showed enlarged ventricles, and thalamic nuclei such as the medial geniculate, substantia nigra, and periaqueductal gray area volumes, as well as right dorsal hippocampus. In contrast, *APOE33HN* showed larger olfactory areas, ventral pallidum, left ventral hippocampus, cerebellum, and brain stem regions, as well as areas of the corpus callosum. Interestingly, the hippocampus showed different laterality in terms of volume for these two genotypes.

When comparing *APOE22HN* versus *APOE44HN*, we observed for the *APOE22HN* mice clusters indicating larger hippocampus, anterior pretectal nuclei, periaqueductal grey area, and mammillary bodies/hypothalamus. In the *APOE44HN* mice, we observed clusters with larger volume in the olfactory areas, corpus callosum, cingulate cortex, striatum and cerebellum.

When comparing *APOE33HN* versus *APOE44HN* mice, we observed that *APOE33HN* mice had a larger dorsal tegmentum and cerebellum. *APOE44HN* mice had a larger corpus callosum, habenula, left S1 Table, and lateral ventricles.

A sex-based comparison indicated males had larger olfactory areas, motor cortex, entorhinal cortex, and visual cortex; in contrast females had larger corpus callosum, bed nucleus of stria terminalis, ventral pallidum, caudate putamen, amygdala, vestibular nuclei, and pons.

## Discussion and conclusions

This work has established a robust pipeline for brain region segmentation and morphometric comparison in mouse brains scanned with hybrid micro-CT. Our hybrid micro-CT scanning reduces scan time by more than an hour per brain compared to previous MRI protocols for mouse brain morphometry [1, 37] while also providing higher spatial resolution (22 vs. 43 μm). Furthermore, we have shown that hybrid iterative reconstruction reduces artifacts from the PCD while retaining its benefits in terms of contrast and spatial resolution. We created a PCD CT mouse brain atlas and showed its qualitative and quantitative similarity to the prior DW MRI atlas. Finally, we demonstrated successful transfer of region labels from this PCD CT atlas to our 26 mouse brains scanned with hybrid micro-CT via SAMBA registration and used these results for group comparisons by genotype and sex.

The use of PCD images in our mouse brain segmentation pipeline offers several benefits. In addition to having superior contrast to noise ratio and spatial resolution to the EID, the PCD enables material decomposition in mouse brains by providing simultaneous multi-energy imaging. As we have shown in Fig 9, the iodine material map of a mouse brain image can provide higher contrast between structures than PCD images. Decomposition could also be effective in ensuring that the stained brain only appears in the iodine map while other obstructive elements such as skull fragments only appear in a calcium map. However, further evaluation is needed to assess if the iodine material map from our reconstructions can be used to improve SAMBA segmentation.

Our use of EID scanning and hybrid iterative reconstruction of mouse brains is effective in producing PCD images with minimal artifacts for SAMBA processing. However, we have demonstrated that EID scanning requires an additional 2 hours per brain, and hybrid iterative reconstruction results in spatial resolution that is superior to EID only but inferior to PCD only. Future work should explore alternate methods for correction of PCD projections that do not require EID scanning. Such corrections would further improve the advantages of PCD mouse brain imaging in terms of scan time and spatial resolution. Furthermore, while our prior studies on the imaging system used for brain scans demonstrated that hybrid iterative reconstruction results in fewer ring artifacts and more accurate and less noisy material decomposition than PCD iterative reconstruction [41], image quality may be further improved by more explicit corrections of PCD spectral distortions such as supervised deep learning with labels from multi-energy EID scans [51].

Our region-based and voxel-based analyses found more differences between male and female mice than between genotypes. Although our ANOVA comparing total brain volume by sex and by genotype both failed to reject the null hypothesis, the boxplots are consistent with our expectation of smaller brain volume in females [50] and in *APOE44HN* genotype [37]. At the regional level, only sex differences survived the FDR correction. Our findings of sex differences in *APOE* models are corroborated by differences reported in C57 mouse models for the amygdala, striatum, and olfactory bulb [50]. While this prior study also reported changes in other regions based on deformation magnitudes, including the hippocampus, these changes were not reflected in the regional volumes for our study. However, we found significant differences by sex in the entorhinal cortex, and in voxel-based analyses for the olfactory bulbs and thalamus. Differences with sex were also found in the bed nucleus of stria terminalis, which has often been reported as sexually dimorphic in humans [52], sensory areas such as the visual cortex, and in the vestibular nuclei, which have been reported as sexually dimorphic in rats [53]. These results suggest that a larger mouse population is needed to allow analysis of genotype by sex interactions.

Although the differences were much more pronounced by sex, our voxel-based pairwise comparisons by genotype did have some noteworthy findings. These analyses indicated differences in the hippocampus, which is consistent with previous MRI studies comparing the *APOE33HN* and *APOE44HN* mouse models [37]. However, our voxel-based analysis only found larger hippocampus for *APOE22HN* mice relative to *APOE44HN*, which is interesting because the *APOE2* allele is considered protective in aging and AD. Genotype differences in the hippocampus are notable because this region is often reported as a biomarker for neurodegenerative diseases in humans [54] and has been associated with spatial memory deficits in mice [47].

While it is noteworthy that the results of our region-based and voxel-based analyses on PCD micro-CT brain images had some similarities to findings from earlier MRI studies, we acknowledge that acquiring MRI and PCD CT scans of the same brains in future work will make comparison easier and allow us to better characterize the strengths and weaknesses of our PCD CT approach. In addition, future studies on PCD CT mouse brains should investigate the effect of interventions such as exercise, a high fat diet, or novel pharmaceutical treatments for AD. Nevertheless, we have demonstrated feasibility of our new PCD CT pipeline for mouse brain analysis, and enabled future large-scale studies of mouse models of AD risk with faster scanning time.

## Supporting information

S1 Table Size distribution of brain regions by genotype. For each genotype, the sizes of all 330 brain regions are expressed as a percentage of the total brain volume (last row).
(DOCX)
