# Supplementary material for: High-resolution hybrid micro-CT imaging pipeline for mouse brain region segmentation and volumetric morphometry
Source: PLoS One. 2024 May 23;19(5):e0303288. doi: 10.1371/journal.pone.0303288 (PMC11115241; doi:10.1371/journal.pone.0303288)
Supplement: S1 Table — For each genotype, the sizes of all 330 brain regions are expressed as a percentage of the total brain volume (last row). (DOCX) [file pone.0303288.s001.docx]

**S1 Table. Size distribution of brain regions by genotype.** For each genotype, the sizes of all 330 brain regions are expressed as a percentage of the total brain volume (last row).

| **Brain Region** | ***APOE22HN* mean** | ***APOE22HN* std dev** | ***APOE33HN* mean** | ***APOE33HN* std dev** | ***APOE44HN***  **mean** | ***APOE44HN***  **std dev** |
| --- | --- | --- | --- | --- | --- | --- |
| Left Cingulate Cortex, Area 24a | 0.1545 | 0.0591 | 0.1257 | 0.0274 | 0.1599 | 0.0463 |
| Left Cingulate Cortex, Area 24a prime | 0.0319 | 0.0113 | 0.0308 | 0.0088 | 0.0319 | 0.0078 |
| Left Cingulate Cortex, Area 24b | 0.137 | 0.0329 | 0.1297 | 0.0303 | 0.1379 | 0.0367 |
| Left Cingulate Cortex, Area 24b prime | 0.0389 | 0.0049 | 0.0421 | 0.0074 | 0.0395 | 0.0045 |
| Left Cingulate Cortex, Area 29a | 0.0483 | 0.0106 | 0.047 | 0.0041 | 0.0554 | 0.0152 |
| Left Cingulate Cortex, Area 29b | 0.0287 | 0.0048 | 0.0271 | 0.0018 | 0.0259 | 0.0018 |
| Left Cingulate Cortex, Area 29c | 0.0621 | 0.0132 | 0.0625 | 0.0089 | 0.0643 | 0.0075 |
| Left Cingulate Cortex, Area 30 | 0.2589 | 0.0329 | 0.2747 | 0.0137 | 0.2557 | 0.0233 |
| Left Cingulate Cortex, Area 32 | 0.265 | 0.0553 | 0.2173 | 0.0231 | 0.2478 | 0.0423 |
| Left Primary Auditory Cortex | 0.1632 | 0.01 | 0.1625 | 0.0103 | 0.1667 | 0.0094 |
| Left Secondary Auditory Cortex, Dorsal Part | 0.1824 | 0.0142 | 0.185 | 0.017 | 0.188 | 0.0088 |
| Left Secondary Auditory Cortex, Ventral Part | 0.1842 | 0.0098 | 0.1919 | 0.0131 | 0.1943 | 0.0067 |
| Left Dorsolateral Orbital Cortex | 0.0925 | 0.0251 | 0.0806 | 0.0158 | 0.0791 | 0.0134 |
| Left Frontal Cortex, Area 3 | 0.1649 | 0.0074 | 0.1587 | 0.0148 | 0.1613 | 0.0077 |
| Left Frontal Association Cortex | 0.9726 | 0.2654 | 0.8779 | 0.2415 | 0.8428 | 0.1433 |
| Left Insular Cortex | 0.8297 | 0.0967 | 0.8149 | 0.1175 | 0.7977 | 0.0809 |
| Left Lateral Orbital Cortex | 0.2333 | 0.0245 | 0.2317 | 0.0158 | 0.2315 | 0.016 |
| Left Lateral Parietal Association Cortex | 0.0281 | 0.0035 | 0.0293 | 0.0028 | 0.0279 | 0.0027 |
| Left Primary Motor Cortex | 0.8723 | 0.0151 | 0.8335 | 0.0604 | 0.8779 | 0.0461 |
| Left Secondary Motor Cortex | 0.7746 | 0.0726 | 0.7559 | 0.0766 | 0.7611 | 0.0466 |
| Left Medial Orbital Cortex | 0.1476 | 0.0309 | 0.1225 | 0.0146 | 0.1388 | 0.0265 |
| Left Medial Parietal Association Cortex | 0.0523 | 0.006 | 0.0575 | 0.0048 | 0.0532 | 0.0046 |
| Left Parietal Cortex, Posterior Area, Rostral Part | 0.0088 | 0.0011 | 0.0089 | 0.001 | 0.0085 | 0.0011 |
| Left Primary Somatosensory Cortex | 0.0843 | 0.0073 | 0.0894 | 0.0061 | 0.0879 | 0.0042 |
| Left Primary Somatosensory Cortex, Barrel Field | 0.9319 | 0.0414 | 0.9625 | 0.0312 | 0.9763 | 0.0295 |
| Left Primary Somatosensory Cortex, Dysgranular Zone | 0.154 | 0.0081 | 0.1555 | 0.0061 | 0.1616 | 0.0085 |
| Left Primary Somatosensory Cortex,Forelimb Region | 0.4199 | 0.0221 | 0.4127 | 0.0141 | 0.4304 | 0.0231 |
| Left Primary Somatosensory Cortex, Hindlimb Region | 0.3713 | 0.0155 | 0.3697 | 0.0118 | 0.3755 | 0.0151 |
| Left Primary Somatosensory Cortex, Jaw Region | 0.3184 | 0.0168 | 0.3031 | 0.0364 | 0.3116 | 0.0121 |
| Left Primary Somatosensory Cortex, Shoulder Region | 0.0138 | 0.001 | 0.0153 | 0.0006 | 0.0146 | 0.0008 |
| Left Primary Somatosensory Cortex, Trunk Region | 0.0758 | 0.0079 | 0.0809 | 0.006 | 0.0783 | 0.0062 |
| Left Primary Somatosensory Cortex, Upper Lip Region | 0.4977 | 0.0202 | 0.497 | 0.041 | 0.5018 | 0.0169 |
| Left Secondary Somatosensory Cortex | 0.5875 | 0.0409 | 0.6088 | 0.0707 | 0.6041 | 0.0328 |
| Left Temporal Association Cortex | 0.3536 | 0.0223 | 0.3549 | 0.035 | 0.3642 | 0.0108 |
| Left Primary Visual Cortex | 0.0683 | 0.0076 | 0.0712 | 0.0046 | 0.0675 | 0.0063 |
| Left Primary Visual Cortex, Binocular Area | 0.2211 | 0.0174 | 0.2181 | 0.0121 | 0.2191 | 0.0177 |
| Left Primary Visual Cortex, Monocular Area | 0.5036 | 0.0605 | 0.4992 | 0.0503 | 0.4895 | 0.0546 |
| Left Secondary Visual Cortex,Lateral Area | 0.2782 | 0.0175 | 0.2728 | 0.0191 | 0.2813 | 0.0187 |
| Left Secondary Visual Cortex, Mediolateral Area | 0.1415 | 0.017 | 0.1511 | 0.0146 | 0.1428 | 0.0176 |
| Left Secondary Visual Cortex, Mediomedial Area | 0.2232 | 0.0301 | 0.2382 | 0.0234 | 0.2269 | 0.029 |
| Left Ventral Orbital Cortex | 0.1561 | 0.0542 | 0.1537 | 0.0596 | 0.1685 | 0.0468 |
| Left Caudomedial Entorhinal Cortex | 0.5166 | 0.0666 | 0.4733 | 0.0588 | 0.4943 | 0.0465 |
| Left Dorsal Intermediate Entorhinal Cortex | 0.2007 | 0.0302 | 0.2038 | 0.0413 | 0.2027 | 0.0248 |
| Left Dorsolateral Entorhinal Cortex | 0.3204 | 0.0456 | 0.3081 | 0.0477 | 0.313 | 0.0289 |
| Left Medial Entorhinal Cortex | 0.0873 | 0.0114 | 0.0901 | 0.0198 | 0.0909 | 0.0136 |
| Left Ventral Intermediate Entorhinal Cortex | 0.1364 | 0.0239 | 0.1489 | 0.0336 | 0.1484 | 0.0232 |
| Left Claustrum | 0.023 | 0.0035 | 0.0253 | 0.004 | 0.0237 | 0.003 |
| Left Dorsal Claustrum | 0.003 | 0.0005 | 0.0039 | 0.0006 | 0.0031 | 0.0005 |
| Left Posterolateral Cortical Amygdaloid Area | 0.1169 | 0.0386 | 0.1239 | 0.0243 | 0.1261 | 0.0273 |
| Left Ventral Claustrum | 0.0114 | 0.0015 | 0.0143 | 0.0019 | 0.0123 | 0.0019 |
| Left Hippocampus | 2.4347 | 0.1239 | 2.388 | 0.1328 | 2.3483 | 0.1535 |
| Left Dorsal Tenia Tecta | 0.0609 | 0.0195 | 0.0532 | 0.0096 | 0.0608 | 0.0149 |
| Left Ectorhinal Cortex | 0.3349 | 0.032 | 0.3304 | 0.042 | 0.3389 | 0.0148 |
| Left Parasubiculum | 0.1469 | 0.0143 | 0.1387 | 0.0209 | 0.149 | 0.0129 |
| Left Perirhinal Cortex | 0.2432 | 0.0245 | 0.2369 | 0.0309 | 0.2379 | 0.0109 |
| Left Presubiculum | 0.019 | 0.0035 | 0.0188 | 0.0037 | 0.0187 | 0.004 |
| Left Piriform Cortex | 4.6297 | 0.2833 | 4.7893 | 0.4407 | 4.941 | 0.4875 |
| Left Amygdalopiriform Transition Area | 0.0561 | 0.0102 | 0.0532 | 0.0078 | 0.0549 | 0.008 |
| Left Hypothalamus | 0.9379 | 0.1726 | 0.8827 | 0.1293 | 0.9606 | 0.104 |
| Left Preoptic Telencephalon | 0.4803 | 0.037 | 0.5243 | 0.1 | 0.5339 | 0.0673 |
| Left Subthalamic Nucleus | 0.0268 | 0.004 | 0.0217 | 0.0032 | 0.0226 | 0.0039 |
| Left Septum | 0.6496 | 0.0475 | 0.6545 | 0.037 | 0.6905 | 0.0393 |
| Left Globus Pallidus | 0.1634 | 0.0294 | 0.179 | 0.0191 | 0.1756 | 0.0288 |
| Left Striatum | 2.1859 | 0.2365 | 2.2554 | 0.2917 | 2.3664 | 0.3034 |
| Left Amygdala | 0.6898 | 0.098 | 0.8092 | 0.1219 | 0.7923 | 0.1122 |
| Left Accumbens | 0.2599 | 0.0277 | 0.2772 | 0.0375 | 0.2983 | 0.0395 |
| Left Bed Nucleus of the Stria Terminalis | 0.0777 | 0.006 | 0.0692 | 0.0083 | 0.0702 | 0.0079 |
| Left Ventral Pallidum | 0.0743 | 0.0144 | 0.092 | 0.0212 | 0.0964 | 0.0277 |
| Left Periaquaductal Grey | 0.3816 | 0.0579 | 0.3227 | 0.0581 | 0.3051 | 0.0305 |
| Left Anterior Pretectal Nucleus | 0.0731 | 0.005 | 0.078 | 0.0075 | 0.0742 | 0.0096 |
| Left Ventral Tegmental Area | 0.0273 | 0.0047 | 0.0243 | 0.0022 | 0.0253 | 0.0031 |
| Left Thalamus Rest | 0.9181 | 0.0904 | 0.876 | 0.0342 | 0.8703 | 0.0474 |
| Left Ventral Thalamic Nuclei | 0.2915 | 0.0223 | 0.3118 | 0.0127 | 0.2936 | 0.0399 |
| Left Latero Dorsal Nucleus of Thalamus | 0.0555 | 0.0058 | 0.0562 | 0.0048 | 0.0536 | 0.0048 |
| Left Medial Geniculate Nucleus | 0.1702 | 0.0048 | 0.1606 | 0.0089 | 0.1483 | 0.0113 |
| Left Lateral Geniculate Nucleus | 0.0736 | 0.0094 | 0.0751 | 0.0053 | 0.0752 | 0.01 |
| Left Zona Incerta | 0.2923 | 0.0374 | 0.3224 | 0.037 | 0.3 | 0.0374 |
| Left Reticular Nucleus of Thalamus | 0.2884 | 0.0278 | 0.3226 | 0.0305 | 0.3024 | 0.0378 |
| Left Subbrachial Nucleus and Peripeduncular Nucleus | 0.0345 | 0.0078 | 0.0301 | 0.0028 | 0.0288 | 0.0049 |
| Left Deep Mesencephalic Nuclei | 0.2131 | 0.0226 | 0.2167 | 0.0283 | 0.2179 | 0.0238 |
| Left Superior Colliculus | 1.1134 | 0.0403 | 1.0917 | 0.0474 | 1.044 | 0.0279 |
| Left Inferior Colliculus | 0.8178 | 0.03 | 0.8121 | 0.0266 | 0.7692 | 0.0417 |
| Left Substantia Nigra | 0.1705 | 0.0265 | 0.1611 | 0.0194 | 0.1486 | 0.0289 |
| Left Red Nucleus Parvicellular | 0.0077 | 0.0021 | 0.0067 | 0.0004 | 0.0067 | 0.0016 |
| Left Midbrain Reticular Nucleus | 0.5404 | 0.0668 | 0.5682 | 0.0411 | 0.5114 | 0.0562 |
| Left Rostral Linear Nucleus | 0.0109 | 0.0016 | 0.0097 | 0.001 | 0.0097 | 0.0018 |
| Left Cuneiform Nucleus | 0.1147 | 0.0065 | 0.1051 | 0.0162 | 0.1034 | 0.014 |
| Left Precuneiform Nucleus | 0.0742 | 0.0064 | 0.0645 | 0.0072 | 0.0664 | 0.0068 |
| Left Brain Stem Rest | 0.7642 | 0.0765 | 0.7957 | 0.0974 | 0.7293 | 0.0648 |
| Left Interpeduncular Nucleus | 0.0202 | 0.0015 | 0.0211 | 0.0022 | 0.0198 | 0.002 |
| Left Cerebellar Cortex | 5.1892 | 0.144 | 5.3007 | 0.2048 | 5.0773 | 0.3019 |
| Left Dentate (Lateral) Nucleus of Cerebellum | 0.024 | 0.003 | 0.026 | 0.0044 | 0.0222 | 0.0031 |
| Left Interposed Nucleus of Cerebellum | 0.0427 | 0.0017 | 0.0375 | 0.0031 | 0.0367 | 0.0045 |
| Left Fastigial Medial Dorsolateral Nucleus of Cerebellum | 0.0066 | 0.001 | 0.0049 | 0.0012 | 0.0049 | 0.001 |
| Left Fastigial Medial Nucleus of Cerebellum | 0.1078 | 0.0072 | 0.0953 | 0.0148 | 0.094 | 0.0079 |
| Left Ventral Lateral Lemniscus Nucleus | 0.0191 | 0.0025 | 0.0172 | 0.0024 | 0.0185 | 0.0029 |
| Left Parabrachial Nucleus | 0.0513 | 0.0077 | 0.0528 | 0.0052 | 0.0516 | 0.0087 |
| Left Parabrachial Medial Nucleus and Koelliker Fuse Nucleus | 0.0944 | 0.0103 | 0.1082 | 0.0123 | 0.0937 | 0.0151 |
| Left Parvicellular Reticular Nucleus and Principal Sensory Trigeminal Nucleus | 0.3862 | 0.0361 | 0.4456 | 0.0359 | 0.447 | 0.0335 |
| Left Central Gray | 0.2641 | 0.0604 | 0.2824 | 0.048 | 0.2604 | 0.0334 |
| Left Pedunculotegmental, Medial Paralemniscial, and Supratrigemnial Nuclei | 0.0413 | 0.0101 | 0.0437 | 0.007 | 0.0451 | 0.0098 |
| Left Motor Root of Trigeminal Nerve | 0.008 | 0.0008 | 0.0095 | 0.0015 | 0.0097 | 0.0014 |
| Left Trigeminal Motor Nucleus | 0.0167 | 0.0068 | 0.0192 | 0.0059 | 0.0193 | 0.0045 |
| Left Pontine Reticular Nucleus | 0.6333 | 0.0972 | 0.6748 | 0.0688 | 0.6038 | 0.122 |
| Left Raphe Nucleus | 0.044 | 0.0084 | 0.0444 | 0.0072 | 0.037 | 0.0096 |
| Left Trigeminal Sensory Nucleus | 0.0732 | 0.0147 | 0.074 | 0.0077 | 0.0726 | 0.0055 |
| Left Dorsal Tegmentum | 0.16 | 0.0184 | 0.1717 | 0.0139 | 0.1515 | 0.0166 |
| Left Tegmental Nucleus | 0.0066 | 0.001 | 0.0058 | 0.0017 | 0.0058 | 0.0008 |
| Left Cochlear Nucleus | 0.0892 | 0.0122 | 0.0951 | 0.0149 | 0.0929 | 0.0108 |
| Left Pontine Nucleus | 0.1323 | 0.0287 | 0.1455 | 0.0329 | 0.1248 | 0.0245 |
| Left Reticulotegmental Nucleus of Pons | 0.0422 | 0.0057 | 0.0475 | 0.0075 | 0.0418 | 0.009 |
| Left Olivary Complex | 0.2269 | 0.041 | 0.2378 | 0.0511 | 0.2302 | 0.0406 |
| Left Pontine Reticular Nucleus | 0.2383 | 0.0559 | 0.2774 | 0.0386 | 0.258 | 0.0411 |
| Left Spinal Trigeminal Nucleus | 0.4709 | 0.0984 | 0.4836 | 0.0731 | 0.4519 | 0.0968 |
| Left Vestibular Nuclei | 0.2697 | 0.0507 | 0.2863 | 0.039 | 0.2767 | 0.029 |
| Left Gigantocellular Reticular Nucleus | 0.7009 | 0.1171 | 0.8293 | 0.1543 | 0.7648 | 0.0938 |
| Left Cuneate Nucleus | 0.0587 | 0.0141 | 0.062 | 0.0118 | 0.0576 | 0.0083 |
| Left Anterior Commisure | 0.1284 | 0.0327 | 0.1482 | 0.0381 | 0.1514 | 0.0349 |
| Left Optic Tracts | 0.1831 | 0.0189 | 0.172 | 0.0162 | 0.1755 | 0.018 |
| Left Fimbria | 0.2138 | 0.0125 | 0.2259 | 0.0128 | 0.2269 | 0.0196 |
| Left Corpus Callosum | 1.2297 | 0.0594 | 1.2396 | 0.0579 | 1.3133 | 0.0551 |
| Left Fornix | 0.024 | 0.0016 | 0.0261 | 0.0014 | 0.0263 | 0.0018 |
| Left Stria Terminalis | 0.0232 | 0.0049 | 0.0257 | 0.0024 | 0.0255 | 0.0037 |
| Left Cingulum | 0.1302 | 0.0226 | 0.1384 | 0.0165 | 0.1495 | 0.0202 |
| Left Lateral Olfactory Tract | 0.1307 | 0.0275 | 0.1197 | 0.0151 | 0.1242 | 0.0147 |
| Left Ventral Hippocampal Commissure | 0.0097 | 0.0011 | 0.0088 | 0.0005 | 0.0097 | 0.0005 |
| Left Internal Capsule | 0.2982 | 0.0376 | 0.3397 | 0.04 | 0.3182 | 0.0421 |
| Left Fasciculus Retroflexus | 0.0032 | 0.0006 | 0.0033 | 0.0005 | 0.0033 | 0.0005 |
| Left Stria Medularis | 0.0232 | 0.0024 | 0.0228 | 0.0023 | 0.0237 | 0.0026 |
| Left Posterior Commissure | 0.017 | 0.0017 | 0.0172 | 0.0018 | 0.0166 | 0.0019 |
| Left Brachium of Superior Colliculus | 0.0264 | 0.003 | 0.0227 | 0.0033 | 0.0213 | 0.0024 |
| Left Cerebral Peduncle | 0.3271 | 0.0447 | 0.2926 | 0.0205 | 0.3103 | 0.045 |
| Left Lateral Lemniscus | 0.1026 | 0.0132 | 0.0974 | 0.0119 | 0.0973 | 0.0145 |
| Left Spinal Trigeminal Nerve | 0.3216 | 0.0506 | 0.3498 | 0.0286 | 0.3523 | 0.0349 |
| Left Pyramidal Tract | 0.1259 | 0.0146 | 0.1409 | 0.027 | 0.1316 | 0.0227 |
| Left Vestibulocochlear Nerve | 0.0154 | 0.0038 | 0.0155 | 0.0024 | 0.0169 | 0.0041 |
| Left Facial Nerve | 0.0072 | 0.0021 | 0.0079 | 0.0021 | 0.0076 | 0.0016 |
| Left Longitudinal Fasciculus of Pons | 0.0074 | 0.0023 | 0.0097 | 0.0023 | 0.0071 | 0.0018 |
| Left Medial Longitudinal Fasciculus and Tectospinal Tract | 0.3931 | 0.0525 | 0.4561 | 0.0604 | 0.3906 | 0.0532 |
| Left Spinocerebellar Tract | 0.1784 | 0.0496 | 0.2308 | 0.1484 | 0.1761 | 0.0768 |
| Left Medial Lemniscus | 0.0242 | 0.0043 | 0.0216 | 0.0027 | 0.0217 | 0.0042 |
| Left Ventral Spinocerebellar Tract | 0.1099 | 0.0179 | 0.112 | 0.0146 | 0.1066 | 0.0135 |
| Left Middle Cerebellar Peduncle | 0.2415 | 0.0569 | 0.2904 | 0.0873 | 0.2597 | 0.0282 |
| Left Superior Cerebellar Peduncle | 0.0996 | 0.016 | 0.1023 | 0.0065 | 0.0994 | 0.0109 |
| Left Inferior Cerebellar Peduncle | 0.0519 | 0.0054 | 0.0591 | 0.0057 | 0.0571 | 0.0049 |
| Left Cerebellar White Matter | 0.8531 | 0.0631 | 0.9078 | 0.0845 | 0.8626 | 0.0716 |
| Left Lateral Ventricle | 0.9948 | 0.2341 | 0.7641 | 0.0892 | 0.8535 | 0.1609 |
| Left Cingulate Cortex, Area 25 | 0.0083 | 0.0016 | 0.0083 | 0.0006 | 0.0096 | 0.002 |
| Left Dorsal Acustic Stria | 0.0055 | 0.0014 | 0.0074 | 0.0027 | 0.0063 | 0.0009 |
| Left Postsubiculum | 0.1049 | 0.0097 | 0.101 | 0.0168 | 0.1029 | 0.0132 |
| Left Ventricular System 4thVentricle | 0.1914 | 0.0344 | 0.1958 | 0.029 | 0.1846 | 0.0158 |
| Left Microcellular Tegmental Nucleus | 0.1473 | 0.0243 | 0.1256 | 0.0093 | 0.1308 | 0.0194 |
| Left Pretectal Nucleus | 0.0063 | 0.0008 | 0.0063 | 0.001 | 0.0064 | 0.0009 |
| Left Latero Dorsal Thalamic Nucleus Ventro Lateral | 0.0164 | 0.0033 | 0.0181 | 0.0022 | 0.0176 | 0.002 |
| Left Latero Posterior Nuclei of Thalamus | 0.0349 | 0.0061 | 0.0354 | 0.0036 | 0.0368 | 0.005 |
| Left Anterior Thalamic Nuclei | 0.112 | 0.0074 | 0.1115 | 0.0049 | 0.1061 | 0.004 |
| Left Red Nucleus Magnocellular | 0.0109 | 0.002 | 0.0104 | 0.0008 | 0.0093 | 0.0023 |
| Left Pararubral Nucleus | 0.0045 | 0.0011 | 0.0039 | 0.0007 | 0.0035 | 0.001 |
| Left Retro Rubral Field | 0.0605 | 0.0108 | 0.0543 | 0.0052 | 0.0525 | 0.0086 |
| Left CSF | 0.1339 | 0.0219 | 0.1182 | 0.0106 | 0.1343 | 0.02 |
| Left Intermediate Reticular Nucleus | 0.1475 | 0.0363 | 0.1839 | 0.0422 | 0.1817 | 0.0274 |
| Left PHD PaMP Post and Lateral Hypothalamus | 0.0791 | 0.0162 | 0.0709 | 0.0101 | 0.0682 | 0.0107 |
| Left Prerubral Forel | 0.0058 | 0.0015 | 0.0059 | 0.0011 | 0.0054 | 0.0009 |
| Left PVG of Hypothalamus | 0.0033 | 0.0006 | 0.0031 | 0.0006 | 0.0027 | 0.0005 |
| Left BLA Basalateral Amygdala | 0.0989 | 0.0182 | 0.1167 | 0.0256 | 0.1267 | 0.0456 |
| Right Cingulate Cortex, Area 24a | 0.1542 | 0.0591 | 0.1186 | 0.03 | 0.1526 | 0.0471 |
| Right Cingulate Cortex, Area 24a prime | 0.0275 | 0.0102 | 0.0258 | 0.0066 | 0.0293 | 0.0093 |
| Right Cingulate Cortex, Area 24b | 0.1314 | 0.0264 | 0.1259 | 0.0357 | 0.1387 | 0.0334 |
| Right Cingulate Cortex, Area 24b prime | 0.0363 | 0.004 | 0.0385 | 0.0075 | 0.0387 | 0.0056 |
| Right Cingulate Cortex, Area 29a | 0.0462 | 0.0123 | 0.0476 | 0.0106 | 0.0519 | 0.0137 |
| Right Cingulate Cortex, Area 29b | 0.025 | 0.0037 | 0.025 | 0.0031 | 0.0251 | 0.0022 |
| Right Cingulate Cortex, Area 29c | 0.0588 | 0.0126 | 0.0599 | 0.0093 | 0.0616 | 0.009 |
| Right Cingulate Cortex, Area 30 | 0.2484 | 0.0234 | 0.2546 | 0.014 | 0.2557 | 0.0207 |
| Right Cingulate Cortex, Area 32 | 0.2613 | 0.0593 | 0.2158 | 0.0316 | 0.2462 | 0.0415 |
| Right Primary Auditory Cortex | 0.1667 | 0.0189 | 0.1671 | 0.0031 | 0.1693 | 0.0124 |
| Right Secondary Auditory Cortex, Dorsal Part | 0.1908 | 0.0236 | 0.1955 | 0.0099 | 0.1919 | 0.0207 |
| Right Secondary Auditory Cortex, Ventral Part | 0.1863 | 0.0169 | 0.1962 | 0.0093 | 0.1936 | 0.0109 |
| Right Dorsolateral Orbital Cortex | 0.0749 | 0.0117 | 0.0756 | 0.0121 | 0.0793 | 0.016 |
| Right Frontal Cortex, Area 3 | 0.1569 | 0.0114 | 0.16 | 0.0173 | 0.1583 | 0.0068 |
| Right Frontal Association Cortex | 0.8953 | 0.2432 | 0.893 | 0.2581 | 0.8011 | 0.1388 |
| Right Insular Cortex | 0.832 | 0.0834 | 0.8415 | 0.1244 | 0.8531 | 0.112 |
| Right Lateral Orbital Cortex | 0.2299 | 0.0151 | 0.2281 | 0.021 | 0.2387 | 0.0235 |
| Right Lateral Parietal Association Cortex | 0.0262 | 0.0025 | 0.0281 | 0.0018 | 0.027 | 0.0022 |
| Right Primary Motor Cortex | 0.8664 | 0.0381 | 0.8451 | 0.0434 | 0.8532 | 0.0573 |
| Right Secondary Motor Cortex | 0.764 | 0.0864 | 0.7325 | 0.0585 | 0.744 | 0.068 |
| Right Medial Orbital Cortex | 0.1993 | 0.0444 | 0.1738 | 0.0322 | 0.1873 | 0.0339 |
| Right Medial Parietal Association Cortex | 0.0514 | 0.0052 | 0.0541 | 0.0037 | 0.053 | 0.003 |
| Right Parietal Cortex, Posterior Area, Rostral Part | 0.0073 | 0.0011 | 0.0078 | 0.0006 | 0.0075 | 0.0009 |
| Right Primary Somatosensory Cortex | 0.0868 | 0.0089 | 0.0914 | 0.0036 | 0.0896 | 0.0067 |
| Right Primary Somatosensory Cortex, Barrel Field | 0.9954 | 0.0586 | 1.0194 | 0.0584 | 1.0115 | 0.0656 |
| Right Primary Somatosensory Cortex, Dysgranular Zone | 0.1575 | 0.0113 | 0.1547 | 0.0108 | 0.1568 | 0.0158 |
| Right Primary Somatosensory Cortex,Forelimb Region | 0.4353 | 0.027 | 0.4252 | 0.0228 | 0.4326 | 0.0402 |
| Right Primary Somatosensory Cortex, Hindlimb Region | 0.3785 | 0.0167 | 0.3708 | 0.0182 | 0.3715 | 0.0258 |
| Right Primary Somatosensory Cortex, Jaw Region | 0.3127 | 0.0227 | 0.3239 | 0.0289 | 0.3214 | 0.0207 |
| Right Primary Somatosensory Cortex, Shoulder Region | 0.0132 | 0.0016 | 0.0141 | 0.0009 | 0.0134 | 0.0012 |
| Right Primary Somatosensory Cortex, Trunk Region | 0.0774 | 0.0082 | 0.0838 | 0.0044 | 0.0793 | 0.0051 |
| Right Primary Somatosensory Cortex, Upper Lip Region | 0.5054 | 0.0265 | 0.5441 | 0.0492 | 0.524 | 0.0469 |
| Right Secondary Somatosensory Cortex | 0.6123 | 0.0436 | 0.6593 | 0.039 | 0.6264 | 0.0494 |
| Right Temporal Association Cortex | 0.3568 | 0.0338 | 0.3705 | 0.0277 | 0.3697 | 0.026 |
| Right Primary Visual Cortex | 0.0685 | 0.0062 | 0.0721 | 0.0042 | 0.0698 | 0.0062 |
| Right Primary Visual Cortex, Binocular Area | 0.2311 | 0.0216 | 0.2301 | 0.0091 | 0.2281 | 0.0191 |
| Right Primary Visual Cortex, Monocular Area | 0.5279 | 0.0567 | 0.5299 | 0.0477 | 0.5101 | 0.0539 |
| Right Secondary Visual Cortex,Lateral Area | 0.2921 | 0.0256 | 0.2885 | 0.0101 | 0.2885 | 0.0216 |
| Right Secondary Visual Cortex, Mediolateral Area | 0.1426 | 0.0106 | 0.1528 | 0.0169 | 0.1457 | 0.0141 |
| Right Secondary Visual Cortex, Mediomedial Area | 0.2299 | 0.0166 | 0.2403 | 0.0252 | 0.2378 | 0.0253 |
| Right Ventral Orbital Cortex | 0.169 | 0.0678 | 0.1478 | 0.048 | 0.1754 | 0.0458 |
| Right Caudomedial Entorhinal Cortex | 0.4958 | 0.0355 | 0.5022 | 0.0694 | 0.4954 | 0.055 |
| Right Dorsal Intermediate Entorhinal Cortex | 0.2262 | 0.0354 | 0.22 | 0.0426 | 0.197 | 0.0278 |
| Right Dorsolateral Entorhinal Cortex | 0.33 | 0.0456 | 0.3297 | 0.062 | 0.3065 | 0.0366 |
| Right Medial Entorhinal Cortex | 0.0905 | 0.0138 | 0.0929 | 0.0239 | 0.0852 | 0.0137 |
| Right Ventral Intermediate Entorhinal Cortex | 0.1581 | 0.0269 | 0.159 | 0.0391 | 0.1433 | 0.025 |
| Right Claustrum | 0.02 | 0.0038 | 0.0214 | 0.0026 | 0.0223 | 0.0036 |
| Right Dorsal Claustrum | 0.0016 | 0.0002 | 0.002 | 0.0002 | 0.0019 | 0.0002 |
| Right Posterolateral Cortical Amygdaloid Area | 0.159 | 0.0144 | 0.1518 | 0.03 | 0.1556 | 0.0306 |
| Right Ventral Claustrum | 0.0078 | 0.0007 | 0.0091 | 0.0013 | 0.0093 | 0.0013 |
| Right Hippocampus | 2.495 | 0.0989 | 2.3975 | 0.1302 | 2.3827 | 0.1146 |
| Right Dorsal Tenia Tecta | 0.0576 | 0.0176 | 0.0509 | 0.0107 | 0.0608 | 0.0161 |
| Right Ectorhinal Cortex | 0.3397 | 0.0355 | 0.3558 | 0.0502 | 0.3419 | 0.0214 |
| Right Parasubiculum | 0.1505 | 0.0184 | 0.1451 | 0.0234 | 0.1522 | 0.0102 |
| Right Perirhinal Cortex | 0.2452 | 0.0215 | 0.2496 | 0.038 | 0.2415 | 0.0207 |
| Right Presubiculum | 0.0199 | 0.0046 | 0.018 | 0.003 | 0.0192 | 0.003 |
| Right Piriform Cortex | 4.488 | 0.3596 | 4.8058 | 0.554 | 5.1841 | 0.623 |
| Right Amygdalopiriform Transition Area | 0.0647 | 0.0078 | 0.0603 | 0.0099 | 0.0587 | 0.0104 |
| Right Hypothalamus | 0.9059 | 0.1474 | 0.8384 | 0.0773 | 0.9667 | 0.133 |
| Right Preoptic Telencephalon | 0.5872 | 0.0827 | 0.5658 | 0.0472 | 0.5914 | 0.0313 |
| Right Subthalamic Nucleus | 0.0262 | 0.0046 | 0.0213 | 0.0016 | 0.0227 | 0.0032 |
| Right Septum | 0.6835 | 0.059 | 0.6605 | 0.0221 | 0.6817 | 0.031 |
| Right Globus Pallidus | 0.1837 | 0.0091 | 0.1888 | 0.0164 | 0.1914 | 0.0184 |
| Right Striatum | 2.521 | 0.1768 | 2.308 | 0.1552 | 2.4815 | 0.3074 |
| Right Amygdala | 0.9195 | 0.0738 | 0.8646 | 0.0754 | 0.9055 | 0.0496 |
| Right Accumbens | 0.3049 | 0.0347 | 0.2968 | 0.0303 | 0.3297 | 0.022 |
| Right Bed Nucleus of the Stria Terminalis | 0.0839 | 0.009 | 0.0747 | 0.0062 | 0.0777 | 0.0137 |
| Right Ventral Pallidum | 0.1025 | 0.0102 | 0.1074 | 0.0287 | 0.1141 | 0.014 |
| Right Periaquaductal Grey | 0.365 | 0.0381 | 0.3026 | 0.0504 | 0.3007 | 0.029 |
| Right Anterior Pretectal Nucleus | 0.0735 | 0.0073 | 0.0712 | 0.006 | 0.071 | 0.0074 |
| Right Ventral Tegmental Area | 0.0281 | 0.0055 | 0.0236 | 0.0032 | 0.0227 | 0.0034 |
| Right Thalamus Rest | 0.9163 | 0.0232 | 0.8615 | 0.0403 | 0.8855 | 0.0722 |
| Right Ventral Thalamic Nuclei | 0.3217 | 0.0274 | 0.3083 | 0.0248 | 0.3065 | 0.0334 |
| Right Latero Dorsal Nucleus of Thalamus | 0.0574 | 0.0047 | 0.0522 | 0.004 | 0.0526 | 0.0052 |
| Right Medial Geniculate Nucleus | 0.1755 | 0.0229 | 0.1584 | 0.0113 | 0.1582 | 0.011 |
| Right Lateral Geniculate Nucleus | 0.0725 | 0.0094 | 0.0736 | 0.0067 | 0.0693 | 0.0091 |
| Right Zona Incerta | 0.3072 | 0.0224 | 0.3153 | 0.0212 | 0.3258 | 0.0233 |
| Right Reticular Nucleus of Thalamus | 0.3125 | 0.0198 | 0.3033 | 0.031 | 0.3034 | 0.0236 |
| Right Subbrachial Nucleus and Peripeduncular Nucleus | 0.0293 | 0.0048 | 0.029 | 0.0046 | 0.0266 | 0.0025 |
| Right Deep Mesencephalic Nuclei | 0.2031 | 0.0252 | 0.204 | 0.0289 | 0.2056 | 0.0189 |
| Right Superior Colliculus | 1.0927 | 0.0452 | 1.0789 | 0.0441 | 1.0425 | 0.0566 |
| Right Inferior Colliculus | 0.832 | 0.0371 | 0.8051 | 0.021 | 0.7732 | 0.0463 |
| Right Substantia Nigra | 0.1889 | 0.0218 | 0.1605 | 0.0138 | 0.1643 | 0.0166 |
| Right Red Nucleus Parvicellular | 0.0053 | 0.0011 | 0.0051 | 0.0006 | 0.0054 | 0.0012 |
| Right Midbrain Reticular Nucleus | 0.5401 | 0.061 | 0.5587 | 0.0426 | 0.5357 | 0.0543 |
| Right Rostral Linear Nucleus | 0.0078 | 0.0013 | 0.007 | 0.0011 | 0.0072 | 0.0014 |
| Right Cuneiform Nucleus | 0.1138 | 0.0096 | 0.1005 | 0.0098 | 0.1012 | 0.0091 |
| Right Precuneiform Nucleus | 0.0667 | 0.0089 | 0.0731 | 0.0081 | 0.0682 | 0.0094 |
| Right Brain Stem Rest | 0.7497 | 0.0651 | 0.7208 | 0.0689 | 0.6953 | 0.0633 |
| Right Interpeduncular Nucleus | 0.018 | 0.0012 | 0.0179 | 0.0022 | 0.0188 | 0.0026 |
| Right Cerebellar Cortex | 5.2697 | 0.2341 | 5.4182 | 0.2225 | 5.1819 | 0.3216 |
| Right Dentate (Lateral) Nucleus of Cerebellum | 0.0237 | 0.0034 | 0.0258 | 0.0048 | 0.0218 | 0.0017 |
| Right Interposed Nucleus of Cerebellum | 0.0434 | 0.003 | 0.0392 | 0.0045 | 0.0396 | 0.005 |
| Right Fastigial Medial Dorsolateral Nucleus of Cerebellum | 0.0051 | 0.0008 | 0.0038 | 0.0009 | 0.004 | 0.0009 |
| Right Fastigial Medial Nucleus of Cerebellum | 0.1072 | 0.0103 | 0.0958 | 0.0108 | 0.0949 | 0.0104 |
| Right Ventral Lateral Lemniscus Nucleus | 0.0193 | 0.0024 | 0.0182 | 0.0018 | 0.0167 | 0.0023 |
| Right Parabrachial Nucleus | 0.0567 | 0.0066 | 0.0532 | 0.0062 | 0.0498 | 0.0068 |
| Right Parabrachial Medial Nucleus and Koelliker Fuse Nucleus | 0.1079 | 0.0151 | 0.1066 | 0.0075 | 0.1065 | 0.0124 |
| Right Parvicellular Reticular Nucleus and Principal Sensory Trigeminal Nucleus | 0.4136 | 0.0337 | 0.438 | 0.0203 | 0.4263 | 0.0246 |
| Right Central Gray | 0.3065 | 0.0638 | 0.2791 | 0.0398 | 0.2814 | 0.0269 |
| Right Pedunculotegmental, Medial Paralemniscial, and Supratrigemnial Nuclei | 0.0527 | 0.0035 | 0.0496 | 0.0054 | 0.0481 | 0.0055 |
| Right Motor Root of Trigeminal Nerve | 0.0081 | 0.001 | 0.009 | 0.0022 | 0.0083 | 0.0017 |
| Right Trigeminal Motor Nucleus | 0.0253 | 0.0073 | 0.0206 | 0.004 | 0.0201 | 0.004 |
| Right Pontine Reticular Nucleus | 0.6975 | 0.0445 | 0.6995 | 0.0494 | 0.6564 | 0.0516 |
| Right Raphe Nucleus | 0.0385 | 0.0089 | 0.04 | 0.0056 | 0.0348 | 0.005 |
| Right Trigeminal Sensory Nucleus | 0.068 | 0.0082 | 0.0695 | 0.0041 | 0.0618 | 0.0083 |
| Right Dorsal Tegmentum | 0.1586 | 0.0115 | 0.1659 | 0.0088 | 0.1608 | 0.0103 |
| Right Tegmental Nucleus | 0.0054 | 0.0008 | 0.0049 | 0.0011 | 0.0048 | 0.0007 |
| Right Cochlear Nucleus | 0.0827 | 0.0118 | 0.0825 | 0.0091 | 0.0844 | 0.0083 |
| Right Pontine Nucleus | 0.1286 | 0.0194 | 0.1344 | 0.0149 | 0.1367 | 0.0157 |
| Right Reticulotegmental Nucleus of Pons | 0.0498 | 0.0145 | 0.0477 | 0.0058 | 0.0441 | 0.0059 |
| Right Olivary Complex | 0.1987 | 0.0241 | 0.1943 | 0.0303 | 0.2071 | 0.0284 |
| Right Pontine Reticular Nucleus | 0.2671 | 0.0336 | 0.2727 | 0.0399 | 0.2596 | 0.0277 |
| Right Spinal Trigeminal Nucleus | 0.4622 | 0.1268 | 0.4543 | 0.0966 | 0.4215 | 0.0876 |
| Right Vestibular Nuclei | 0.2996 | 0.0336 | 0.284 | 0.019 | 0.2837 | 0.0129 |
| Right Gigantocellular Reticular Nucleus | 0.7437 | 0.0976 | 0.7734 | 0.1077 | 0.7447 | 0.075 |
| Right Cuneate Nucleus | 0.0559 | 0.0111 | 0.052 | 0.0082 | 0.0552 | 0.0066 |
| Right Anterior Commisure | 0.1559 | 0.0283 | 0.1481 | 0.0223 | 0.1706 | 0.0372 |
| Right Optic Tracts | 0.1631 | 0.0201 | 0.157 | 0.0131 | 0.1541 | 0.0071 |
| Right Fimbria | 0.2354 | 0.0165 | 0.2274 | 0.0057 | 0.2289 | 0.0096 |
| Right Corpus Callosum | 1.2388 | 0.0766 | 1.209 | 0.0429 | 1.2918 | 0.0489 |
| Right Fornix | 0.0229 | 0.0015 | 0.0239 | 0.0017 | 0.024 | 0.0029 |
| Right Stria Terminalis | 0.0257 | 0.0029 | 0.0219 | 0.0022 | 0.022 | 0.002 |
| Right Cingulum | 0.1393 | 0.035 | 0.1331 | 0.0174 | 0.1473 | 0.021 |
| Right Lateral Olfactory Tract | 0.1165 | 0.0081 | 0.111 | 0.0076 | 0.121 | 0.0132 |
| Right Ventral Hippocampal Commissure | 0.0085 | 0.001 | 0.0079 | 0.0008 | 0.0084 | 0.0007 |
| Right Internal Capsule | 0.3899 | 0.0269 | 0.3739 | 0.0453 | 0.387 | 0.0236 |
| Right Fasciculus Retroflexus | 0.0021 | 0.0003 | 0.0021 | 0.0004 | 0.002 | 0.0002 |
| Right Stria Medularis | 0.0214 | 0.0027 | 0.0222 | 0.0032 | 0.0227 | 0.0044 |
| Right Posterior Commissure | 0.0148 | 0.0015 | 0.0158 | 0.0024 | 0.0143 | 0.0013 |
| Right Brachium of Superior Colliculus | 0.0282 | 0.0053 | 0.0239 | 0.0037 | 0.0207 | 0.0041 |
| Right Cerebral Peduncle | 0.2906 | 0.0289 | 0.2742 | 0.0223 | 0.2829 | 0.0234 |
| Right Lateral Lemniscus | 0.1147 | 0.0118 | 0.1063 | 0.0104 | 0.0978 | 0.0117 |
| Right Spinal Trigeminal Nerve | 0.2747 | 0.024 | 0.2918 | 0.0262 | 0.292 | 0.0262 |
| Right Pyramidal Tract | 0.1345 | 0.0306 | 0.1204 | 0.0115 | 0.1413 | 0.04 |
| Right Vestibulocochlear Nerve | 0.0141 | 0.0037 | 0.0171 | 0.0036 | 0.0152 | 0.0025 |
| Right Facial Nerve | 0.0071 | 0.0022 | 0.0067 | 0.0016 | 0.007 | 0.0013 |
| Right Longitudinal Fasciculus of Pons | 0.006 | 0.0014 | 0.0066 | 0.0005 | 0.007 | 0.0011 |
| Right Medial Longitudinal Fasciculus and Tectospinal Tract | 0.3678 | 0.0466 | 0.4062 | 0.0503 | 0.3675 | 0.0425 |
| Right Spinocerebellar Tract | 0.1566 | 0.0489 | 0.1638 | 0.0472 | 0.1598 | 0.0508 |
| Right Medial Lemniscus | 0.0215 | 0.0008 | 0.0183 | 0.0021 | 0.0188 | 0.0026 |
| Right Ventral Spinocerebellar Tract | 0.1127 | 0.0119 | 0.1088 | 0.0061 | 0.1073 | 0.0081 |
| Right Middle Cerebellar Peduncle | 0.2427 | 0.0298 | 0.2424 | 0.0249 | 0.238 | 0.0342 |
| Right Superior Cerebellar Peduncle | 0.1015 | 0.0114 | 0.0991 | 0.0061 | 0.0955 | 0.0057 |
| Right Inferior Cerebellar Peduncle | 0.0541 | 0.0138 | 0.0605 | 0.0077 | 0.0585 | 0.004 |
| Right Cerebellar White Matter | 0.8395 | 0.0668 | 0.9151 | 0.0732 | 0.8651 | 0.1042 |
| Right Lateral Ventricle | 1.0623 | 0.32 | 0.7578 | 0.1012 | 0.8117 | 0.1766 |
| Right Cingulate Cortex, Area 25 | 0.008 | 0.0018 | 0.0076 | 0.001 | 0.0086 | 0.0012 |
| Right Dorsal Acustic Stria | 0.005 | 0.0016 | 0.0059 | 0.001 | 0.0055 | 0.0005 |
| Right Postsubiculum | 0.1084 | 0.0134 | 0.1047 | 0.0101 | 0.1061 | 0.0097 |
| Right Ventricular System 4thVentricle | 0.1913 | 0.0226 | 0.1979 | 0.0303 | 0.187 | 0.0134 |
| Right Microcellular Tegmental Nucleus | 0.1473 | 0.0151 | 0.1401 | 0.0147 | 0.1365 | 0.0194 |
| Right Pretectal Nucleus | 0.0048 | 0.0009 | 0.0045 | 0.0005 | 0.0047 | 0.0009 |
| Right Latero Dorsal Thalamic Nucleus Ventro Lateral | 0.0173 | 0.0026 | 0.018 | 0.0026 | 0.016 | 0.002 |
| Right Latero Posterior Nuclei of Thalamus | 0.0371 | 0.0051 | 0.0354 | 0.0037 | 0.0337 | 0.0048 |
| Right Anterior Thalamic Nuclei | 0.1271 | 0.0074 | 0.1222 | 0.0083 | 0.1196 | 0.0084 |
| Right Red Nucleus Magnocellular | 0.0092 | 0.0021 | 0.0091 | 0.001 | 0.0089 | 0.0015 |
| Right Pararubral Nucleus | 0.0026 | 0.0005 | 0.0021 | 0.0002 | 0.0021 | 0.0006 |
| Right Retro Rubral Field | 0.0577 | 0.0085 | 0.0546 | 0.0052 | 0.0583 | 0.0059 |
| Right CSF | 0.1286 | 0.0223 | 0.1144 | 0.0093 | 0.1288 | 0.0173 |
| Right Intermediate Reticular Nucleus | 0.1788 | 0.0382 | 0.1808 | 0.0434 | 0.1813 | 0.0228 |
| Right PHD PaMP Post and Lateral Hypothalamus | 0.083 | 0.0073 | 0.0738 | 0.0115 | 0.0706 | 0.0057 |
| Right Prerubral Forel | 0.0054 | 0.0009 | 0.0054 | 0.001 | 0.0058 | 0.0006 |
| Right PVG of Hypothalamus | 0.0023 | 0.0004 | 0.0021 | 0.0004 | 0.0018 | 0.0002 |
| Right BLA Basalateral Amygdala | 0.1187 | 0.02 | 0.1178 | 0.0292 | 0.1275 | 0.0257 |
| Total Brain Volume (mm^3^) | 234.5359 | 20.7935 | 223.9333 | 14.178 | 214.9924 | 21.0347 |
